# Human umbilical cord mesenchymal stem cells and biological scaffolds: application and advances in oral and maxillofacial tissue engineering

**DOI:** 10.3389/fbioe.2026.1970237

**Published:** 2026-10-28

**Authors:** Chuping Hou, Huan Jiang, Ronglin Wang

**Affiliations:** 1 Department of Prosthodontics, Jinan Stomatological Hospital, Jinan, Shandong, China; 2 Jinan Key Laboratory of Oral Tissue Regeneration, Shandong Provincial Key Medical and Health Laboratory of Oral Diseases and Tissue Regeneration, Jinan, Shandong, China; 3 Department of Endodontics, Jinan Stomatological Hospital, Jinan, Shandong, China

**Keywords:** biological scaffolds, human umbilical cord mesenchymal stem cells, maxillofacial, oral, regeneration, tissue engineering

## Abstract

Extensive defects and functional impairments in oral and maxillofacial tissues pose significant clinical challenges, with current standard treatments facing inherent limitations. Tissue engineering, integrating seed cells, scaffolds, and bioactive factors, offers a promising regenerative approach. Human umbilical cord mesenchymal stem cells (hUCMSCs) have emerged as a promising and clinically advantageous cell source due to their robust multi-lineage potency, immunomodulatory capacity, low immunogenicity, and non-invasive procurement. However, their therapeutic efficacy relies on delivery systems that ensure cell retention and fate control. This review provides a comprehensive analysis of hUCMSC-based strategies (including both cellular and exosome-mediated approaches) combined with biological scaffolds for tissue engineering. It details the biological characteristics of hUCMSCs, reviews the design, performance, and functionalization strategies (e.g., surface modification, peptide conjugation, and ionic doping) of key scaffold materials (bioceramics, biologically derived scaffolds, polymers, and composites), and highlights their applications in regenerating oral and maxillofacial bone, cartilage, periodontal, and pulp-dentin tissues. The discussion concludes with an examination of current translational challenges and future perspectives, aiming to serve as a reference for advancing next-generation regenerative therapies.

## 1 Introduction

Oral and maxillofacial soft and hard tissue defects profoundly impact patients’ quality of life. Achieving functional regeneration and effective repair of these damaged tissues remains a core challenge in modern oral medicine (Wei et al., 2024). Currently, autologous tissue transplantation is considered the “gold standard” for tissue reconstruction (Ghezzi et al., 2024). However, these approaches are hindered by limitations such as donor site morbidity, restricted availability, immune rejection, and suboptimal clinical outcomes (Hong et al., 2025; Md et al., 2023). Therefore, the development of a safe, effective, and precisely targeted regenerative strategy remains a critical and unmet challenge.

To address this challenge, tissue engineering has emerged as an innovative strategy in regenerative medicine, offering a promising avenue for regenerating oral and maxillofacial tissues. Its core principle is to engineer a bioactive composite with regenerative potential by strategically combining three key elements: seed cells, biological scaffolds, and bioactive factors (Bahraminasab et al., 2025; Tayanloo-Beik et al., 2023). This approach aims not only to restore the anatomical structure of damaged tissues but also to reconstitute their complex physiological functions (Rosa et al., 2025), thereby presenting a transformative solution for repair and functional rehabilitation. Among seed cells, mesenchymal stem cells (MSCs) have garnered significant attention due to their unique biological properties (Hussein, 2025). MSCs can be isolated from various tissues such as bone marrow, adipose tissue, umbilical cord, and dental pulp (Yordanova et al., 2024). Although bone marrow-derived MSCs (BMSCs) are considered the “gold standard” (Zhang M. et al., 2023), their clinical translation is limited by source availability, invasive harvesting, and donor-site morbidity (Hu et al., 2023). In contrast, human umbilical cord-derived mesenchymal stem cells (hUCMSCs) offer abundant availability, non-invasive procurement, rapid proliferation, low immunogenicity, and minimal ethical concerns (Chen Y. et al., 2025), making them a highly promising candidate for tissue engineering (Wei et al., 2025), with their therapeutic effects mediated not only by the cells themselves but also by their paracrine signaling, particularly via exosomes.

However, the therapeutic potential of stem cells is often limited *in vivo* by challenges such as poor cell survival, inadequate retention at the target site, and difficulty in precisely controlling their activity over time (Wang M. et al., 2023; Wu et al., 2022). To overcome these hurdles, the engineering of cell-scaffold constructs has emerged as a pivotal strategy. An advanced biological scaffold serves not merely as a passive carrier but as a three-dimensional, biomimetic niche that promotes cell adhesion, proliferation, and differentiation while enabling controlled and sustained bioactivity (Venkataiah et al., 2021). This synergistic combination of hUCMSCs with functional scaffolds is therefore critical to unlocking their full regenerative potential.

This review provides a comprehensive and critical analysis of the convergence of biological scaffolds with hUCMSC-based therapeutic strategies, which include both cellular and exosome-mediated approaches, in oral and maxillofacial tissue engineering (Figure 1). To this end, it systematically describes the biological characteristics of hUCMSCs, reviews the design and performance of various scaffold materials used in combination with these strategies, and focuses on the application and recent advances of such hUCMSC-based scaffold systems in regenerating key oral tissues including bone, cartilage, periodontal tissue, and the pulp-dentin complex. Finally, by discussing current translational challenges and future directions, this review aims to provide a foundational reference for the development of next-generation regenerative therapies in this field.

**FIGURE 1:**
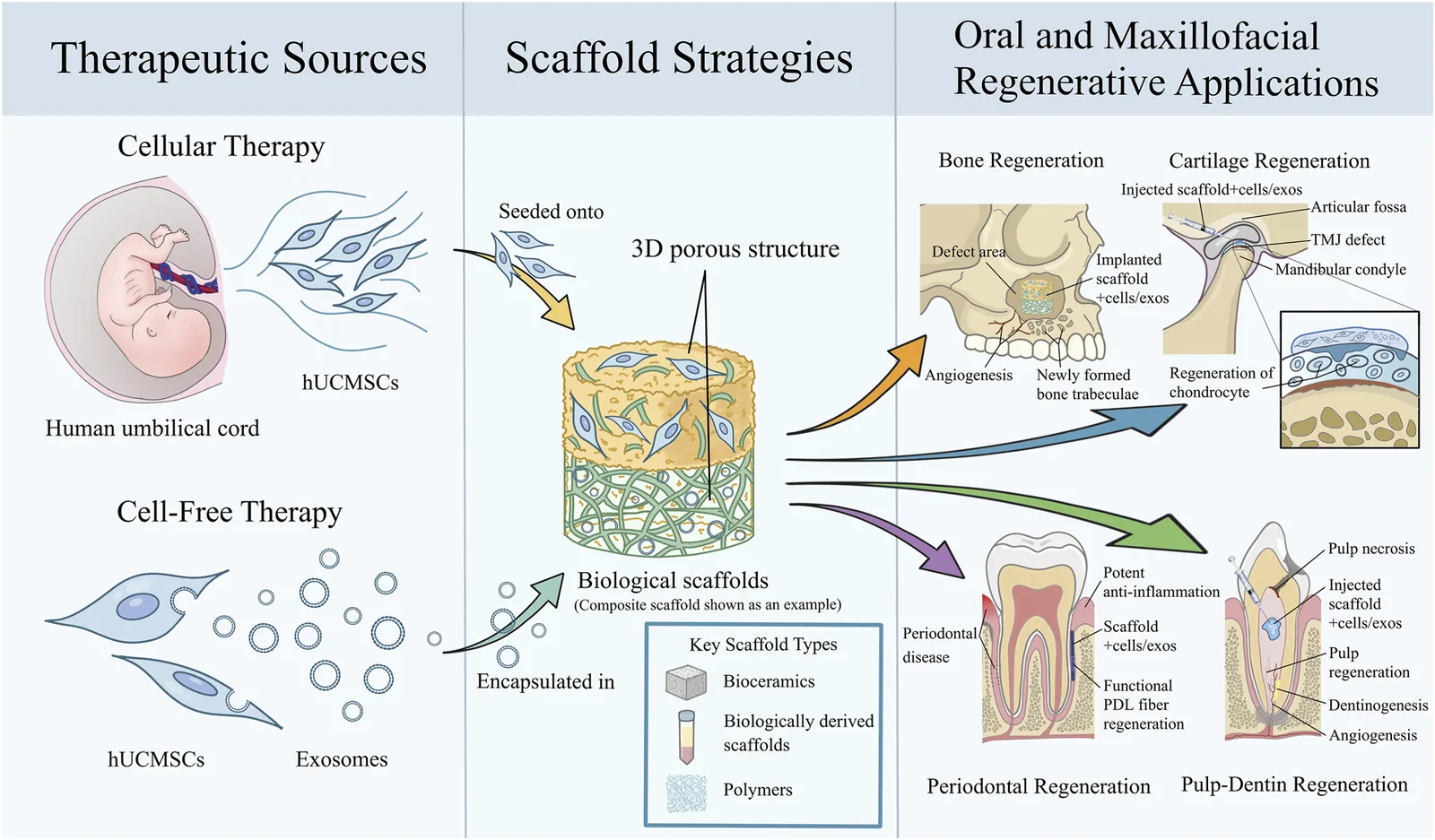
Synergistic strategy for oral and maxillofacial regeneration: hUCMSCs and advanced biological scaffolds. This graphical abstract illustrates the integrative approach of combining human umbilical cord mesenchymal stem cells (hUCMSCs) and their derived exosomes with customized biological scaffolds for functional tissue regeneration. From left to right: therapeutic agents (cellular and cell-free) are sourced from hUCMSCs; they are combined with diverse scaffold strategies (bioceramics, biologically derived scaffolds, polymers, and composites); and applied to regenerate key oral and maxillofacial tissues—bone, cartilage, periodontal complex, and the pulp-dentin complex. The schematic emphasizes the scaffold’s role as an active, instructive platform to deliver, retain, and guide therapeutic agents for multi-tissue restoration. Abbreviations: hUCMSCs (human umbilical cord mesenchymal stem cells); TCP (tricalcium phosphate); 3D (three-dimensional); TMJ (temporomandibular joint).

## 2 Human umbilical cord mesenchymal stem cells

hUCMSCs have emerged as a promising cell source in tissue engineering. Derived from medical waste of newborn umbilical cords, their acquisition involves minimal ethical concerns and offers an abundant source (Kwiecien et al., 2025). Depending on the isolation site, they can be obtained from Wharton’s jelly, perivascular tissues, or umbilical cord blood (Hussein, 2025; Naji et al., 2019). In the literature, cells derived from specific anatomical compartments are often precisely designated, such as Wharton’s jelly-derived mesenchymal stem cells (WJMSCs) and umbilical cord blood-derived mesenchymal stem cells (UCB-MSCs). hUCMSC collection is non-invasive, avoiding donor-site morbidity (Yang H. et al., 2024). hUCMSCs have self-renewal capacity and multidirectional differentiation potential, enabling differentiation into various cell lineages. They also maintain viability and functionality through multiple passages, showing resistance to senescence (Ahani-Nahayati et al., 2022). Additionally, hUCMSCs have low immunogenicity due to low or absent expression of co-stimulatory surface molecules, which reduces the likelihood of immune rejection. Thus, allogeneic transplantation of hUCMSCs typically does not require immunosuppressive therapy (Ahani-Nahayati et al., 2022; Zhu et al., 2023). These features underscore the biomedical potential of hUCMSCs in regenerative applications.

Owing to their multifaceted therapeutic mechanisms, hUCMSCs are actively investigated for various diseases. These properties include the capacity to differentiate into multiple cell lineages, thereby promoting tissue regeneration and functional restoration (Fan et al., 2024; Kim et al., 2024). Additionally, hUCMSCs can modulate immune responses by regulating immune cell proliferation and differentiation, as well as influencing cytokine secretion, which further aids in tissue repair (Fan et al., 2024; Sengun et al., 2023; Umer et al., 2023). They also attenuate inflammatory and oxidative stress responses by modulating cytokine production (Chen et al., 2024; Jiang et al., 2024). Moreover, through the regulation of non-coding RNA expression and paracrine signaling, hUCMSCs indirectly influence target genes and promote tissue regeneration (Chen et al., 2024; Kacham et al., 2021; Ruan et al., 2019). Notably, their paracrine effects are partly mediated by the release of extracellular vesicles such as exosomes, which deliver bioactive molecules (Gao et al., 2024). Collectively, these mechanisms not only explain their broad therapeutic potential but, more importantly, provide the theoretical foundation for their application in scaffold-based regenerative strategies, including cell-free approaches utilizing the hUCMSC secretome (Gao et al., 2024) (Figure 2).

**FIGURE 2:**
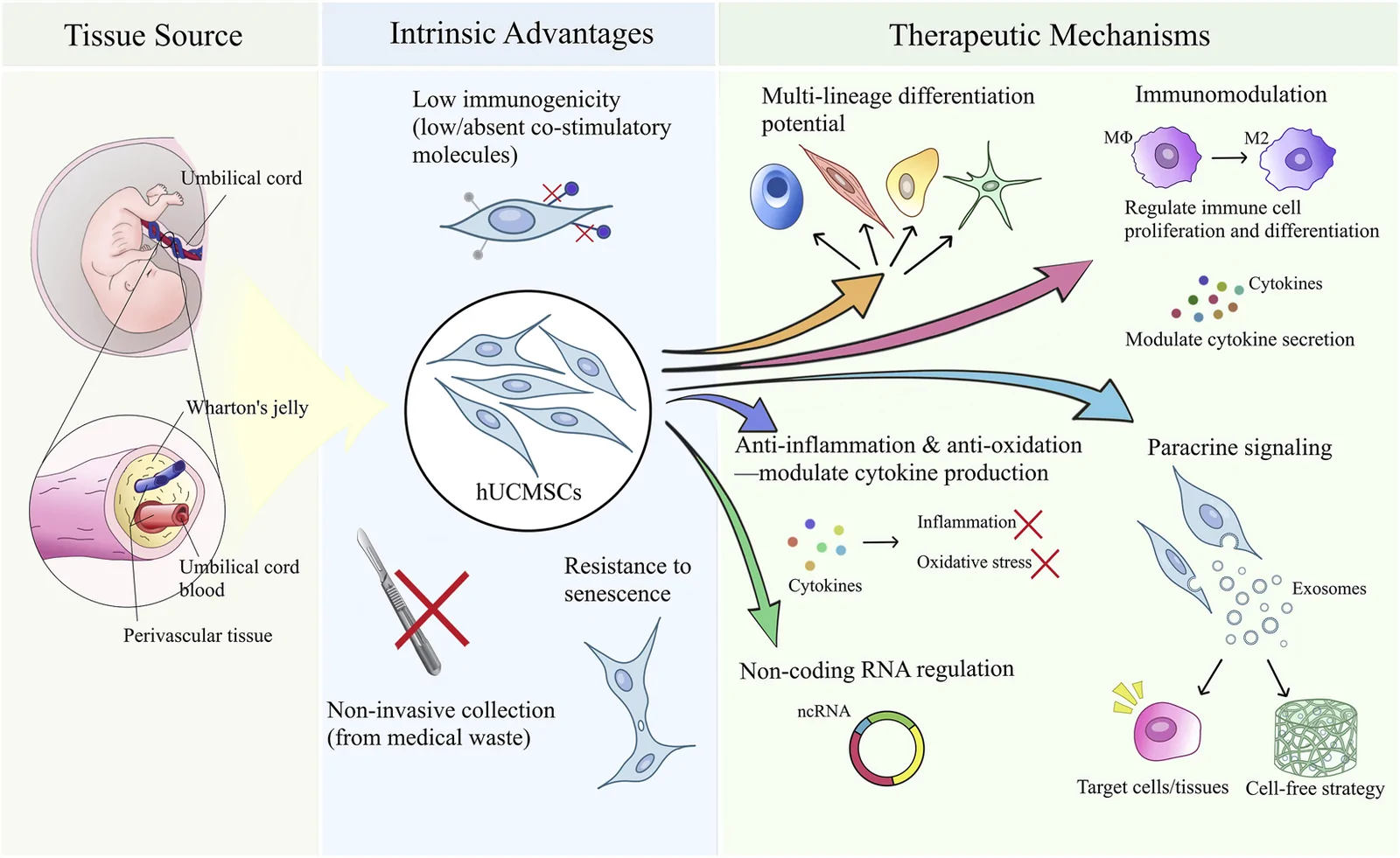
Overview of hUCMSC characteristics. Left panel: hUCMSCs are isolated from Wharton’s jelly, perivascular tissue, and umbilical cord blood. Middle panel: Intrinsic advantages: non-invasive collection, low immunogenicity, and resistance to senescence. Right panel: Therapeutic mechanisms include multi-lineage differentiation, immunomodulation (Mφ→M2, cytokine modulation), anti-inflammation/anti-oxidation, paracrine signaling (exosomes delivering bioactive molecules) enabling cell-free strategies, and non-coding RNA regulation. Abbreviations: hUCMSCs (human umbilical cord mesenchymal stem cells); Mφ (macrophage); M2 (M2-polarized macrophage); ncRNA (non-coding RNA).

It should be noted that the advantages of hUCMSCs are primarily practical and translational, rather than universally superior. Their osteogenic, angiogenic, and chondrogenic capacities are generally considered comparable to those of hBMSCs in many studies (Wang et al., 2009; Wang P et al., 2015; Wen et al., 2013), while oral-derived stem cells such as differentiation of dental pulp stem cells (DPSCs) and periodontal ligament stem cells (PDLSCs) may retain tissue-specific differentiation preferences and functional differences that could favor periodontal and pulp-dentin regeneration (Das and Sloan, 2023; Kadkhoda et al., 2024). A systematic comparison is provided in Table 1. Nevertheless, given their accessibility, immunogenicity profile, and scalability, hUCMSCs remain a highly practical cell source for scaffold-based oral and maxillofacial tissue engineering.

**TABLE 1:** Comparison of biological characteristics of hUCMSCs and other mesenchymal stem cells (BMSCs, DPSCs, PDLSCs).

Feature	hUCMSCs/UC-MSCs	BMSCs	DPSCs	PDLSCs	Key references
Source accessibility	High	Low	Moderate	Moderate	(Das and Sloan, 2023; Kadkhoda et al., 2024; Wen et al., 2013)
Procurement invasiveness	Non-invasive	Invasive	Invasive (requires tooth extraction)	Invasive (requires tooth extraction)	(Das and Sloan, 2023)
Proliferation rate	High	Moderate	High	High	(Das and Sloan, 2023; Kadkhoda et al., 2024; Wen et al., 2013)
Immunogenicity	Low	Moderate	Low	Low	(Das and Sloan, 2023; Kadkhoda et al., 2024; Tang et al., 2019; Yang et al., 2025; Zhu et al., 2023)
Osteogenic potential	Comparable to or slightly lower than BMSCs	Gold standard	Moderate	Higher than UCMSCs	(Gholami et al., 2023; Kadkhoda et al., 2024; Wen et al., 2013)
Odontogenic/periodontal potential	Moderate; enhanced by co-culture	Low	High	High	(Das and Sloan, 2023; Huang et al., 2020; Kadkhoda et al., 2024; Shang et al., 2017)
Immunomodulation	High; low immunogenicity	Moderate	Moderate to High	Moderate	( Anzalone et al., 2010; Geng et al., 2021 ; Kadkhoda et al., 2024 ; Shang et al., 2017 ; Zhai et al., 2024 )

In this context, hUCMSCs have been extensively investigated for oral and maxillofacial soft and hard tissue repair and regeneration. In bone and cartilage regeneration, hUCMSCs possess a gene expression profile similar to that of hBMSCs, supporting their potential in repairing extensive bone defects and regenerating temporomandibular joint cartilage (Zhu et al., 2023). Regarding periodontal tissue regeneration, research indicates that under specific inductive conditions, hUCMSCs can differentiate into human periodontal ligament cells and achieve functional adhesion to the root surface (George et al., 2015; Heinayati et al., 2025). This offers promising prospects for reconstructing the critical periodontal interface known as the “periodontal bone-ligament complex”. In the field of dental pulp-dentin regeneration, evidence suggests that hUCMSCs can be induced to differentiate into odontoblast-like cells. When co-transplanted with endothelial-differentiated cells, they are capable of forming vascularized dental pulp-like tissue *in vivo* (Zhang et al., 2020a). This highlights the application advantages of hUCMSCs in dental pulp regeneration, particularly in vascular and nerve reconstruction. Furthermore, building on the understanding of hUCMSC paracrine mechanisms, active components such as exosomes have been isolated and incorporated into scaffolds, creating a cell-free therapeutic strategy. This approach has also demonstrated considerable promise in bone, periodontal, dentin, and pulp regeneration.

## 3 Biological scaffold materials combined with hUCMSCs

Although hUCMSCs demonstrate significant therapeutic promise in oral and maxillofacial tissue engineering, a major translational challenge remains: how to efficiently and precisely deliver these cells, retain them at complex three-dimensional defect sites, and sustain a suitable microenvironment for their survival, proliferation, and controlled differentiation (Tayanloo-Beik et al., 2023). Oral and maxillofacial tissue defects present unique anatomical and functional complexities, such as intricate biomechanical loads, irregular geometries, and dynamic microbial and biochemical milieus (Loop et al., 2025; Song et al., 2022). In this context, traditional strategies, such as the local injection of cell suspensions, exhibit significant inherent limitations. These include cell dispersion and loss due to the fluid environment, leading to low local survival rates; the absence of structural support, which hinders the formation of desired tissue architecture; and the inability to replicate the natural three-dimensional framework of the extracellular matrix necessary for guiding organized cellular growth and differentiation (Wang et al., 2022).

To address these challenges, combining hUCMSCs with customized biomaterial scaffolds to form cell-scaffold constructs has become a predominant and necessary direction in contemporary oral and maxillofacial regenerative medicine. These biomaterial scaffolds function not only as delivery vehicles for cells but also as versatile enabling platforms that support and guide the regenerative process. An effective scaffold must maintain three-dimensional mechanical strength and chemical stability to closely mimic the physiological environment of native tissues (Hong et al., 2025). It should possess a porous architecture with an interconnected pore network to facilitate the exchange and transport of nutrients and metabolic waste (Camacho-Alonso et al., 2022; Tan et al., 2024). Biologically derived scaffolds should closely mimic the natural extracellular matrix (ECM) of the target tissue, providing a supportive microenvironment for cell adhesion, proliferation, and differentiation, which is critical for successful tissue regeneration (Bari et al., 2022). Such scaffolds must exhibit high biocompatibility, be non-toxic and non-harmful, and minimize immune reactions at the implantation site (Ju et al., 2024; Kiarashi et al., 2024). Additionally, scaffolds should be biodegradable, with a degradation rate that aligns with the pace of new tissue formation (Khalaf et al., 2022). Furthermore, scaffold materials must exhibit specific functional properties tailored to their intended application. For instance, scaffolds designed for bone reconstruction should possess osteogenic, osteoinductive, or osteoconductive characteristics (Bari et al., 2022; Lee et al., 2021), and ideally facilitate the controlled release of bioactive factors that promote angiogenesis and osteogenesis (Tayanloo-Beik et al., 2023). In contrast, scaffolds intended for nerve injury repair should support neuroregeneration and guide axonal regrowth (Kim J. et al., 2025; Yan et al., 2022). Consequently, the structural design, material composition, and cell-seeding methodology of the biological scaffold are critical determinants of the overall efficacy of the tissue-engineered construct. In recent years, the application of biological scaffold materials in conjunction with hUCMSCs has emerged as an active area of research. This article reviews the biological scaffold materials that have been integrated with hUCMSCs for the regeneration of soft and hard tissues in the oral and maxillofacial region: bioceramic materials, biologically derived scaffolds, polymeric materials, and composite materials (Figure 3). It is noteworthy that these same categories of scaffold materials also serve as effective delivery platforms for hUCMSC-derived exosomes, a cell-free therapeutic strategy. The applications of these exosome-functionalized scaffolds will be discussed in the subsequent sections, focusing on specific tissue regeneration.

**FIGURE 3:**
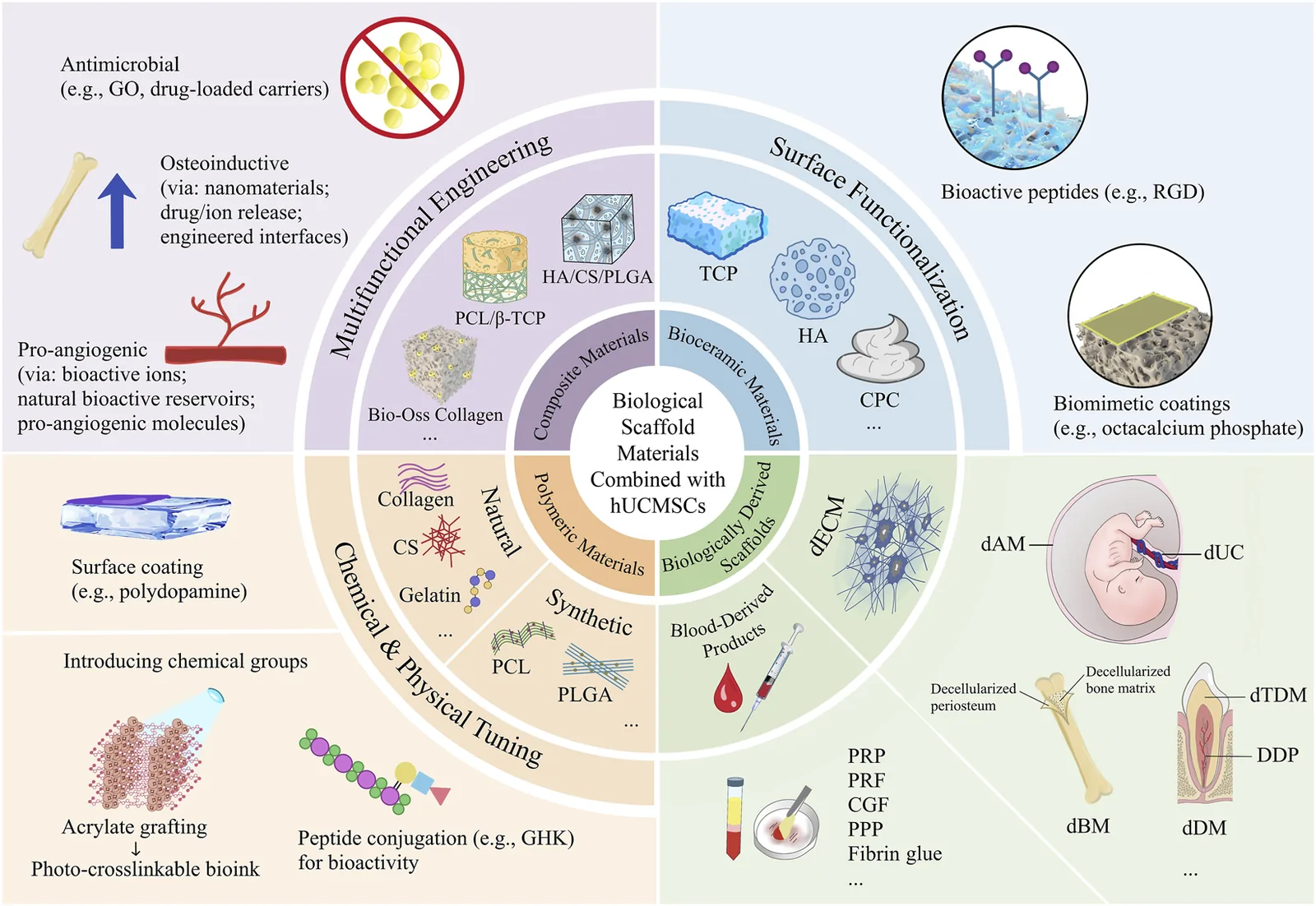
Classification and functionalization of biological scaffold materials combined with hUCMSCs. This schematic diagram categorizes and illustrates the key biological scaffold materials engineered for the delivery and enhancement of hUCMSCs and their derivatives. The concentric circle layout organizes materials into four main classes: Bioceramic Materials, Biologically Derived Scaffolds (detailed in two subclasses: dECM and blood-derived products), Polymeric Materials (natural and synthetic), and Composite Materials. For bioceramic, polymeric, and composite materials, the outermost ring highlights their representative functionalization strategies: surface functionalization for bioceramics, chemical and physical tuning for polymers, and multifunctional engineering for composites. The diagram emphasizes the rational design of scaffolds as versatile platforms that progress from structural supports to bioactive, instructive interfaces for stem cells. Abbreviations: hUCMSCs (human umbilical cord mesenchymal stem cells); TCP (tricalcium phosphate); HA (hydroxyapatite); CPC (calcium phosphate cement); RGD (tripeptide arginyl-glycyl-aspartic acid); dECM (decellularized extracellular matrix); dAM (decellularized amniotic membrane); dUC (decellularized umbilical cord); dBM (bone-derived decellularized matrix); dDM (dental-derived decellularized matrix); dTDM (decellularized tooth dentin matrix); DDP (decellularized dental pulp matrix); PRP (platelet-rich plasma); PRF (platelet-rich fibrin); CGF (concentrated growth factor); PPP (platelet-poor plasma); CS (chitosan); PCL (poly (ε-caprolactone)); PLGA (poly (lactide-co-glycolide)); GHK (glycine-histidine-lysine); β-TCP (β-tricalcium phosphate); GO (graphene oxide).

The discussion within each material category is structured according to its defining characteristics. For synthetically tunable platforms like bioceramics and polymers, we focus on engineering strategies to improve performance. For biologically derived scaffolds, valued for their innate bio-instructive complexity, we organize by tissue origin to highlight their unique niches. This approach ensures a coherent analysis of each scaffold type alongside hUCMSCs.

### 3.1 Bioceramic materials

Bioactive ceramics, including tricalcium phosphate (TCP), hydroxyapatite (HA) and bioactive glasses (Dasan and Chandrasekar, 2023), are the most widely used bioceramics in tissue engineering due to their biocompatibility and osteoconductivity, making them promising scaffolds for bone regeneration with hUCMSCs (Shi et al., 2023; Skliarenko et al., 2024).

However, conventional bioceramics are often fabricated via high-temperature sintering to densify the structure. A major drawback of this process is that it yields scaffolds with high crystallinity and large crystal size, properties that drastically slow their *in vivo* resorption rate, thereby hindering integration with the host bone (Meesuk et al., 2022). Furthermore, their inherent brittleness poses challenges for the repair of thin-walled or irregular bone defects, as mechanical stress after implantation can lead to scaffold fracture, thereby compromising the regenerative outcome (Huang et al., 2022; Liu et al., 2021; Yang Z. et al., 2024). These limitations can restrict their broader applicability in clinical settings.

In this context, researchers have also investigated improved strategies, such as employing three-dimensional (3D) printing technology to precisely control material pore architecture, which subsequently enhances cell adhesion and proliferation (Menshikh et al., 2025). Meanwhile, calcium phosphate cement (CPC) also represents a favorable scaffold option. It is typically composed of tetracalcium phosphate (Ca_4_(PO_4_)_2_O) and dicalcium phosphate anhydrous (CaHPO_4_). In addition to sharing the advantageous properties of conventional bioceramics, CPC exhibits unique characteristics such as self-setting ability, injectability, and plasticity (Xu et al., 2024). This enables it to closely adapt to complex bone cavity geometries, making it especially suitable for repairing and reconstructing bones with irregular contours, such as those in the craniofacial and maxillofacial regions (Chen et al., 2013; Wang P. et al., 2015). Furthermore, the functional modification of bioceramic surfaces can significantly enhance the performance of scaffold materials. For example, the tripeptide arginyl-glycyl-aspartic acid (RGD) serves as a key cell-adhesion motif. Incorporating RGD into CPC can effectively promote cell adhesion to the biomaterial (Chen et al., 2013; Wang P. et al., 2015). One study applied a biomimetic coating containing octacalcium phosphate crystals to 3D-printed HA scaffolds. It was found that these scaffolds supported the proliferation and osteogenic differentiation of hUCMSCs. Moreover, the addition of the octacalcium phosphate biomimetic coating further enhanced the biocompatibility of the bone regeneration scaffold (Meesuk et al., 2022). Given these advantageous and tunable properties, bioceramics have been extensively investigated as scaffolds for delivering hUCMSCs in oral and maxillofacial bone and periodontal regeneration (de Carvalho et al., 2024; Raveau and Jordana, 2020).

### 3.2 Biologically derived scaffolds

The term “biologically derived scaffolds” refers to scaffold materials sourced directly from living organisms or their components, which retain a significant portion of their native supramolecular architecture or multi-component biological activity. Their defining characteristic and primary advantage lies in leveraging the inherent, bio-instructive complexity of their source. This fundamentally distinguishes them from purely synthetic polymers and purified natural polymers. While the latter are derived from nature, they are processed into chemically defined, generic building blocks in which the native higher-order structure is disassembled, and the tissue-specific compositional complexity is significantly reduced or intentionally simplified. In contrast, biologically derived scaffolds encompass a spectrum of materials that leverage this inherent bio-instructive complexity. Primary examples are decellularized extracellular matrix (dECM) and blood-derived products.

#### 3.2.1 Decellularized extracellular matrix

Natural ECM is a three-dimensional framework primarily composed of fibrous proteins (such as collagen type I, II, III, IV, VI, X, and elastin) and glycoproteins (such as proteoglycans, fibronectin, and laminin). It contains abundant cell-recognition sites and diverse protein-adhesion domains, which facilitate the localization and controlled release of growth factors. These growth factors play a crucial role in regulating cellular activities and modulating phenotypic expression (Li et al., 2022). Given its ability to provide essential structural support to cells and release key biological signals that promote tissue morphogenesis, differentiation, and homeostasis, the ECM has become a central focus of research in tissue engineering scaffolds (Ahmed et al., 2024). The dECM is a scaffold material derived from natural human or animal tissues or organs by removing cellular components through physical, chemical, or enzymatic methods. This biological scaffold largely retains the three-dimensional structure, composition, and functional properties of the native ECM, while exhibiting no significant cytotoxicity and eliciting minimal immune rejection or inflammatory responses (Tan et al., 2024; Zeng A. et al., 2022). Consequently, the use of dECM scaffolds facilitates the creation of a repair microenvironment that closely resembles native tissue conditions, thereby enhancing the regeneration of damaged tissues (Ramzan et al., 2022). These scaffolds are categorized based on tissue origin into types such as decellularized amniotic membrane (dAM), decellularized umbilical cord (dUC), bone-derived decellularized matrix (dBM), and dental-derived decellularized matrix (dDM). Their distinct physicochemical signals and biological properties enable their application in repairing diverse tissue injuries (Zhang et al., 2024). In the field of oral and maxillofacial tissue engineering, decellularized matrices further demonstrate considerable potential as scaffold materials when combined with hUCMSCs.

##### 3.2.1.1 Decellularized amniotic membrane

The amniotic membrane (AM), which lines the fetal surface of the placenta, comprises a thick basement membrane and an avascular stroma (Mohan et al., 2017). Its unique ECM composition, rich in diverse collagens and glycoproteins, confers significant biological properties, including potent anti-inflammatory, anti-fibrotic, immunomodulatory, and antimicrobial activities, coupled with inherent low immunogenicity (Le-Buu et al., 2022; Sarı et al., 2019). Studies have shown that the AM possesses inherent properties that promote bone healing and periodontal tissue regeneration (Mohan et al., 2017; Sarı et al., 2019), making it an attractive natural scaffold material for tissue engineering applications. The decellularization of AM not only reduces the risk of infection but also extends the shelf life of the biomaterial, thereby enhancing its feasibility and cost-effectiveness (Ahmed et al., 2024). In terms of the combined application with hUCMSCs, Ahmed et al. demonstrated that dAM possesses a porous surface topography favorable for the attachment and proliferation of hUCMSCs (Ahmed et al., 2024). Building on this, Pham et al. engineered a functional cell sheet by culturing hUCMSCs on a dAM, which exhibited robust biological activity (Le-Buu et al., 2022). Although direct *in vivo* evidence for the application of hUCMSCs seeded on dAM in oral and maxillofacial regeneration is currently limited, the synergistic combination of dAM’s bioactive ECM and the multipotent capacity of hUCMSCs presents a highly promising strategy. Future studies are warranted to validate its efficacy in preclinical models relevant to oral and maxillofacial defects.

##### 3.2.1.2 Decellularized umbilical cord

Human umbilical cord (UC) represents a promising native biological matrix for tissue engineering scaffolds. Its dECM is rich in chondro-conductive components such as collagen, proteoglycans, hyaluronic acid, and inherent growth factors, collectively creating a bioactive microenvironment that supports cell proliferation, matrix synthesis, and tissue remodeling (Ramzan et al., 2022). Evidence suggests that UC-derived scaffolds possess an intrinsic capacity to direct stem cell fate toward a chondrogenic lineage (Safari et al., 2019). In a key study, Ramzan et al. developed a three-dimensional hydrogel from decellularized human umbilical cord (dUC) tissue. Their findings demonstrated that this dUC-derived hydrogel served as a biocompatible and porous three-dimensional framework, effectively supporting the proliferation and chondrogenic differentiation of hUCMSCs *in vitro*, as evidenced by the significant upregulation of key chondrogenic marker genes including SRY-box transcription factor 9 (SOX-9), bone morphogenetic protein 2 (BMP-2), transforming growth factor beta 1 (TGF-β1), and aggrecan (AGGRECAN). Collectively, this work highlights the considerable potential of combining dUC with hUCMSCs for cartilage-like tissue engineering (Ramzan et al., 2022). However, translating this promising *in vitro* evidence into clinical applications for oral and maxillofacial regions, such as temporomandibular joint (TMJ) disc or condylar cartilage regeneration, necessitates further rigorous validation through *in vivo* studies.

##### 3.2.1.3 Bone-derived decellularized matrix

dBM refers to the decellularized matrix derived from bone tissue and its associated structures. Among these structures, decellularized bone matrix and decellularized periosteum are relatively representative materials.

Through decellularization and demineralization, bone tissue can be processed into a porous, deformable scaffold. This scaffold retains its intrinsic osteoconductivity while providing tissue-specific extracellular matrix signals, thereby influencing the behavior of resident and/or seeded cells (Mattioli-Belmonte et al., 2019). Monica et al. seeded hUCMSCs onto a decellularized bone matrix scaffold. They observed that the cells adhered and proliferated well on the scaffold. Furthermore, the scaffold demonstrated the ability to induce differentiation of hUCMSCs *in vitro*, indicating its potential for further testing in in vivo tissue regeneration applications (Mattioli-Belmonte et al., 2019). Sun et al. demonstrated that a composite of decellularized, degreased bovine cancellous bone collagen matrix and hUCMSCs could effectively promote the regenerative repair of alveolar clefts in a rabbit model (Sun X. C. et al., 2023).

Meanwhile, the periosteum is a highly vascularized tissue rich in cells and bioactive factors essential for bone growth and development (Wang J. et al., 2023). Consequently, the decellularized periosteum serves as a highly advantageous scaffold material. Li et al. extracted and prepared the decellularized periosteum-derived hydrogel (dPH) from porcine periosteum tissue, and inoculated hUCMSCs into dPH. They found that this scaffold material could release unique biophysical and biochemical signals, regulating the bioactivities of hUCMSCs, promoting the initial diffusion, migration, and proliferation of hUCMSCs. By accurately simulating the native periosteal microenvironment, the dPH further enhanced the expression of osteogenic-related genes and induced tissue mineralization (Li et al., 2022).

These studies highlight the promising application of dBM, particularly when combined with hUCMSCs, as biological scaffolds in oral and maxillofacial tissue engineering.

##### 3.2.1.4 Dental-derived decellularized matrix

dDM refers to a category of scaffold materials derived from decellularized dental tissues, primarily including decellularized dental pulp matrix (DDP) and decellularized tooth dentin matrix (dTDM). These scaffolds preserve the essential native architecture and biochemical components of the dental extracellular matrix, providing a tissue-specific microenvironment for regeneration.

The DDP retains crucial tissue-specific growth factors and microstructures vital for regenerating the dentin-pulp complex (Bakhtiar et al., 2020). Substantial evidence has demonstrated its efficacy in supporting the viability and proliferation of various stem cell types (Bakhtiar et al., 2020; Matoug-Elwerfelli et al., 2020). For instance, in a significant *in vivo* study, Tan et al. demonstrated that combining WJMSCs with a DDP scaffold could effectively promote dental pulp regeneration (Tan et al., 2024).

Similarly, the dTDM preserves the characteristic microstructure of dentinal tubules, which is critical for guiding dentin regeneration (Chen et al., 2015; Rangiani et al., 2012). Moreover, dTDM retains inherent bioactive factors, such as dentin sialoprotein (DSP) and dentin matrix protein-1 (DMP-1), which play pivotal roles in dentinogenesis and create a conducive microenvironment for odontoblastic differentiation (Liu et al., 2022). Supporting this, Chen et al. demonstrated that human dTDM can provide an odontoblast differentiation microenvironment for hUCMSCs, inducing their differentiation into odontoblast-like cells (Chen et al., 2015).

Collectively, these studies highlight the niche-specific advantages of dDM scaffolds: DDP provides a conducive microenvironment for pulp regeneration, while dTDM uniquely guides odontoblastic differentiation through its preserved tubular microstructure and inherent bioactive factors. This underscores the principle that scaffold sourcing should align with target tissue biology.

Beyond the tissue-specific dECMs detailed in the preceding subsections, several other decellularized matrices have been explored as biologically derived scaffolds in broader tissue engineering contexts and may hold relevance for oral and maxillofacial applications. Examples include dermal matrix and tendon-derived ECM, which has been processed into hydrogels to support hUCMSCs culture in advanced *in vitro* platforms such as microfluidic devices (Bhatt et al., 2022). Notably, some of these materials, such as the commercially available porcine dermal matrix (e.g., Bio-Gide®), have already been utilized in clinical oral regeneration procedures and, as discussed later in this review, have also served as relevant platforms for delivering hUCMSC-derived factors (Li H. et al., 2025). Similarly, Matrigel-derived hydrogel, a soluble basement membrane extract, functions as a bioactive matrix analogous to dECM and has been employed as a scaffold for hUCMSCs in studies focusing on pulp-dentin complex regeneration (Sabeti et al., 2024). While the direct therapeutic application of scaffolds like tendon-derived ECM with hUCMSCs in oral and maxillofacial regeneration models remains less documented, their inclusion here underscores the diversity of available biologically derived scaffold options that merit consideration for future tailored designs.

#### 3.2.2 Blood-derived products

Blood-derived products refer to therapeutic components obtained from human or animal blood through processes primarily involving separation and concentration. This category includes materials such as autologous platelet concentrates (APCs), platelet-poor plasma (PPP), and fibrin glue.

##### 3.2.2.1 Autologous platelet concentrates

APCs are typically prepared by centrifuging venous blood using specific speed/time protocols, with or without the addition of anticoagulants and thrombin. The resulting product is a three-dimensional fibrin scaffold entrapping concentrated platelets and, depending on the protocol, varying numbers of leukocytes (Mijiritsky et al., 2021). Based on their preparation methodology and fibrin architecture, APCs are categorized into successive generations.

Platelet-rich plasma (PRP), the first generation of platelet concentrates, is produced via two-step centrifugation and is commonly activated with bovine thrombin and calcium chloride. Upon activation, it releases key growth factors including TGF-β, platelet-derived growth factor (PDGF), vascular endothelial growth factor (VEGF), and insulin-like growth factor-1 (IGF-1). These factors enhance collagen production, thereby facilitating the regeneration of soft tissue and bone (Nam et al., 2023; Wen et al., 2014). The fibrin matrix of PRP provides a natural, bioactive microenvironment conducive to stem cell survival and function. Studies have shown that when hUCMSCs are directly encapsulated within the PRP hydrogel, they exhibit superior proliferative activity compared to standard culture systems. Furthermore, this PRP scaffold actively enhances the osteogenic differentiation of the embedded hUCMSCs (Wen et al., 2014). Therefore, PRP demonstrates significant potential as a cell-delivery scaffold material in tissue engineering.

The second-generation of APCs is represented by platelet-rich fibrin (PRF). In contrast to PRP, PRF is prepared without the use of anticoagulants, thereby simplifying its production process and theoretically reducing risks associated with exogenous anticoagulants (Malcangi et al., 2023). Furthermore, compared to PRP, PRF provides advantages in terms of cytokine diversity and the kinetics of their release, which underpins its suitability as a candidate scaffold material (Mijiritsky et al., 2021).

Concentrated growth factor (CGF) represents the third generation of APCs. Unlike earlier preparations, CGF membrane formation does not require exogenous activators or polymerization agents, thereby reducing risks of immune reaction and cross-infection (Li et al., 2021). Moreover, CGF exhibits a denser and more robust fibrin matrix than PRF or PRP (Malcangi et al., 2023). This material also possesses notable tensile strength, and its porous architecture effectively shields growth factors from rapid hydrolysis, enabling their sustained and prolonged release (Wang et al., 2024). These properties contribute to its widespread use in clinical treatments and tissue engineering.

Currently, APCs are widely used in tissue regeneration. In the context of oral and maxillofacial tissue engineering, these materials are not merely biological adjuvants but functional scaffold materials in their own right. They have demonstrated significant promise in preclinical and clinical applications, particularly for periodontal tissue repair and dental pulp regeneration (Li et al., 2021; Ramzan et al., 2022; Wen et al., 2014).

##### 3.2.2.2 Platelet-poor plasma

PPP is another blood-derived product, similar in composition to PRP but with a lower platelet concentration (Angelopoulos et al., 2022; Brizuela et al., 2020). Research indicates that PPP and PRP share comparable growth factor profiles and exert similar effects on osteogenic differentiation (Martínez et al., 2016). Consequently, PPP also holds promise as a tissue engineering scaffold material. Interestingly, for specific applications such as dental pulp regeneration, the lower platelet content of PPP may confer an advantage, as high platelet concentrations can sometimes inhibit the proliferation and DPSCs (Angelopoulos et al., 2022). Consequently, PPP-based scaffolds, particularly when combined with hUCMSCs, have emerged as a key research focus for optimizing dental pulp regeneration strategies (Cordero et al., 2020).

##### 3.2.2.3 Fibrin glue

Fibrin glue (or fibrin sealant), a biomaterial that acts as a tissue adhesive, is a candidate material for tissue engineering scaffolds. Fibrin glue is derived from plasma and contains a freeze-dried concentrate of key clotting proteins, including fibrinogen, factor XIII, fibronectin, and thrombin (Ortiz et al., 2021; Sanz-Horta et al., 2023). Thus, while representing a highly processed and standardized form, it fundamentally relies on biologically derived components to recapitulate the core function of a natural fibrin matrix. Fibrin glue serves as a bioactive matrix that supports cell adhesion, viability, proliferation, and differentiation. It is biocompatible, biodegradable, and features a porous architecture that facilitates cell migration, vascular infiltration, and angiogenesis, thereby playing an important role in tissue repair and healing (Ortiz et al., 2022). A study investigating the combination of hUCMSCs and porcine fibrin glue for periodontal tissue healing demonstrated its efficacy, indicating the material’s potential as a scaffold for hUCMSC-based oral and maxillofacial tissue engineering (Li et al., 2014).

### 3.3 Polymeric materials

Polymers are long-chain organic materials held together by covalent bonds (Percival et al., 2024). Those used in tissue engineering can be classified into natural and synthetic polymers based on their source. Commonly employed natural polymers in this field include collagen, gelatin and its derivatives, chitosan (CS), alginate, and hyaluronic acid (Koushik et al., 2023; Liu et al., 2021). These materials, derived from living organisms, exhibit structural similarities to the native ECM, a feature that promotes protein adsorption to their surfaces. Natural polymers typically offer favorable biocompatibility and biodegradability (Koushik et al., 2023; Sufiyan et al., 2024). However, their application can be limited by inadequate mechanical strength, unpredictable degradation profiles, and potential immunogenicity or pathogen risks (Koushik et al., 2023). To address these limitations, researchers have also developed and investigated synthetic polymers. Commonly used synthetic polymers in tissue engineering include polylactide (PLA), poly (ε-caprolactone) (PCL), polyglycolic acid (PGA), and poly (lactide-co-glycolide) (PLGA) (Liu et al., 2021; Percival et al., 2024). Their mechanical strength, modulus, and degradation rate can be tailored to specific application scenarios through chemical modification of their structures (Percival et al., 2024; Sufiyan et al., 2024). This high degree of tunability allows for the rational design of biomaterials. Therefore, strategies such as polymer modification and processing optimization can be employed to meet diverse requirements and ultimately enhance tissue regeneration outcomes.

The strategy of combining polymer scaffolds with hUCMSCs constitutes a common focus of contemporary research. For instance, many studies have employed gelatin as a delivery vehicle for hUCMSCs in the investigation of oral and maxillofacial bone regeneration (Prasetyo et al., 2024; 2025). Zhang et al. selected gelatin-methacrylate (GelMA) hydrogel as the scaffold material and combined it with hUCMSCs engineered to overexpress circCTTN. Their study demonstrated that this hydrogel effectively supports the growth and viability of hUCMSCs (Zhang M. et al., 2023). Zhang et al. utilized type I collagen as a scaffold to encapsulate hUCMSCs and validated its efficacy in promoting dental pulp regeneration (Zhang et al., 2020a; 2020b). Kadam et al. demonstrated through clinical research that a combination of hUCMSCs and PCL scaffolds exhibits a therapeutic effect on gingival recession (Kadam et al., 2019). Collectively, these studies underscore the considerable potential of combining hUCMSCs with rationally designed polymeric scaffolds to advance oral and maxillofacial tissue engineering.

Moving beyond conventional polymers, next-generation polymeric platforms are being engineered to address the inherent limitations of traditional materials, such as batch variability and immunogenicity. A notable example is a 3D-printable hydrogel engineered from recombinant collagen-like protein and functionalized polysaccharide, which offers a defined composition, shear-thinning behavior for bioprinting, and dynamic self-healing properties, thereby offering a promising platform for creating customized scaffolds for stem cell delivery (Jebakumar et al., 2025).

Building upon the exploration of natural and synthetic polymers, strategic surface modification presents a powerful method to enhance the bioactivity of polymeric scaffolds and tailor their interaction with hUCMSCs (Manu et al., 2025). A prime example is a polydopamine-coated, biodegradable shape memory polymer (SMP) scaffold based on cross-linked PCL. The polydopamine coating serves to improve hydrophilicity and provide cell-adhesive motifs (Guda et al., 2024). Furthermore, Kamaraj et al. employed chemical functionalization, which involves grafting acrylate or methacrylate groups onto polyethylene glycol (PEG) and alginate, to create photo-cross-linkable bioinks for 3D bioprinting of hUCMSC-laden constructs. Through controlled variations in polymer composition and crosslinking, they created hydrogels whose tunable stiffness itself functioned as a key osteoinductive cue. Thus, the functionalized scaffold itself provides sufficient osteoinductive instruction to hUCMSCs, showcasing the power of molecular material design in directly programming cell behavior (Kamaraj et al., 2021). Beyond stiffness, the covalent conjugation of bioactive peptides represents another powerful functionalization strategy to endow polymeric scaffolds with specific signaling cues. For instance, Klontzas et al. developed a hydrogel by covalently crosslinking oxidized alginate (alginate dialdehyde; ADA) with the copper-free glycine-histidine-lysine (GHK) peptide through the reaction between the aldehyde groups of ADA and the amine groups of the peptide. This ADA-GHK hydrogel stably retained the peptide within the matrix with minimal release, creating a long-term osteoinductive microenvironment. When encapsulating umbilical cord blood mesenchymal stem cells (UCB-MSCs), this functionalized hydrogel significantly enhanced cell viability, proliferation, and osteogenic differentiation compared to gelatin-containing controls, demonstrating the efficacy of peptide-conjugated polymer networks in directing stem cell fate toward bone formation (Klontzas et al., 2019). Collectively, these findings highlight the synergistic potential of combining advanced, functionally-modified polymer scaffolds with optimized hUCMSC-based strategies.

### 3.4 Composite materials

In tissue engineering, different scaffold materials possess distinct physical, chemical, and biological properties. However, a single material type often has inherent limitations that restrict its application. For example, certain bioceramics can be brittle, exhibit poor fatigue resistance, and present challenges in shaping (Huang et al., 2018). Some polymers suffer from low mechanical strength, a lack of inherent bioactivity, poor hydrophilicity, and an unpredictable degradation rate (de Souza et al., 2024; Li Y et al., 2024). To overcome these limitations, composite scaffolds can be fabricated by integrating two or more distinct materials via physical or chemical means. This approach yields materials with enhanced, tunable properties. Furthermore, the final characteristics of the composite can be precisely adjusted by altering the component ratios. In this context, composite materials are treated as a design strategy rather than a strict material class, encompassing multi-material composites, ion-doped systems, surface-functionalized scaffolds, and bioactive molecule-loaded constructs. Accordingly, this category may overlap with the bioceramic, biologically derived, and polymeric categories described above, depending on the composition and functionalization of the scaffold.

Numerous studies have demonstrated that combining bioactive ceramics with polymers can significantly enhance the mechanical properties of scaffolds. CPC, for instance, is inherently brittle and weak; however, the incorporation of CS has been shown to markedly improve its mechanical strength (Chen et al., 2013). Zhao et al. developed a reinforced CPC scaffold by integrating CS and polyglactin fibers. Their findings revealed that hUCMSCs cultured on these CPC-CS-fiber composites exhibited upregulation of osteogenic markers, such as alkaline phosphatase (ALP) and osteocalcin (OC), compared with cells on non-fibrous CPC controls. The authors attributed this improvement to the greater surface area and roughness provided by the fibers, which facilitate cell attachment and promote osteogenic differentiation (Zhao et al., 2010). PCL, a synthetic aliphatic polyester, degrades slowly and is highly hydrophobic with limited bioactivity, while β-tricalcium phosphate (β-TCP) alone lacks sufficient mechanical strength (Coudane et al., 2022; Ghezzi et al., 2024). By combining PCL and β-TCP into a composite scaffold, researchers have achieved both improved mechanical performance and enhanced cell proliferation (Huang et al., 2018; Nam et al., 2023). In another approach, Wang et al. fabricated an nHA/CS/PLGA composite scaffold to address the individual shortcomings of three materials: nano-hydroxyapatite (nHA), which shows poor biodegradability and low toughness; CS, which exhibits low strength and rapid strength loss in wet conditions; and PLGA, which possesses low mechanical strength and lacks inherent osteogenic bioactivity. This ternary composite not only demonstrated increased mechanical strength but also, through the incorporation of nHA and CS particles, provided greater surface area and roughness within the PLGA matrix. These structural modifications promoted the adhesion and proliferation of hUCMSCs, thereby synergistically enhancing the scaffold’s overall osteogenic repair capacity (Wang et al., 2014).

In addition, several commercial products also represent examples of composite materials. A notable instance is Bio-Oss® Collagen by Geistlich Pharma AG, which consists of 90% Geistlich Bio-Oss® granules and 10% porcine collagen. The small proportion of collagen enhances the material’s handling properties, while the Bio-Oss® component provides a reliable osteoconductive scaffold that supports effective bone formation. The composite is easy to trim, shapeable, and possesses a porous structure, making it a highly suitable scaffold material (Geistlich Pharma, 2026). Kosinski et al. seeded WJMSCs onto Bio-Oss® Collagen scaffolds and observed subsequent cell adhesion and osteogenic differentiation. The resulting cell-scaffold constructs exhibited high cell viability and facilitated the formation of complex osteoblastic structures. Notably, these structures displayed a characteristic loss of intercellular boundaries, indicative of active cell–cell interaction and integration. These findings suggest that the combination of Bio-Oss® Collagen scaffolds with WJMSCs holds significant potential for bone defect repair (Kosinski et al., 2020).

The design of composite scaffolds can be engineered not only to enhance physical and biological properties but also to incorporate specific therapeutic functions that improve the microenvironment for stem cell-based regeneration (Wu et al., 2021; 2020). For instance, integrating components with inherent antimicrobial activity can prevent infection, which is a critical concern in oral and maxillofacial repair, without compromising stem cell viability. To illustrate this approach, Wu et al. first developed an injectable CPC-CS-graphene oxide (GO) composite, leveraging the intrinsic antibacterial properties of GO. This scaffold exhibited high mechanical strength, significant activity against *Staphylococcus aureus*, and supported the adhesion and proliferation of hUCMSCs, confirming its potential as a bioactive platform for stem cell delivery (Wu et al., 2020). Subsequently, the same group refined the design by introducing penicillin-loaded alginate microbeads into a CPC-CS matrix, thereby creating a multifunctional scaffold capable of sustaining hUCMSCs viability while providing localized antibacterial protection. The composite demonstrated mechanical properties comparable to cancellous bone, effective antibacterial performance, and excellent cytocompatibility, highlighting its suitability for applications in complex bone contours (Wu et al., 2021). Together, these studies illustrate a strategic progression in scaffold functionalization aimed at fostering hUCMSC-based regeneration by integrating antimicrobial properties directly into the biomaterial matrix. While these *in vitro* findings are promising, further *in vivo* studies remain essential to validate the scaffolds’ efficacy and translational potential in oral and maxillofacial tissue engineering.

Beyond imparting antimicrobial properties, the functionalization of composite scaffolds can be strategically designed to directly instruct stem cell fate and orchestrate complex regenerative processes, such as osteogenesis and angiogenesis. This functionalization strategy shifts the scaffold’s role from a structural support to a bio-instructive platform, achieved by incorporating specific bioactive molecules, ions, or nanomaterials that deliver precise signaling cues to hUCMSCs (Domingues et al., 2025).

To actively drive osteogenic differentiation, composite scaffolds have been engineered to provide active osteoinductive cues through three primary strategies. The first involves the integration of bioactive nanomaterial interfaces. For instance, incorporating GO into electrospun PCL matrices has been shown to promote the osteogenic differentiation and mineralization of hUCMSCs without exogenous induction, highlighting the potential of nanomaterial-polymer composites as inherently osteoinductive niches (Kashte et al., 2024). The second strategy focuses on constructing sustained-release depots for osteogenic small molecules or ions, such as dexamethasone within collagen-nHA scaffolds (Tanideh et al., 2025) or the flavonoid EGCG encapsulated in CS/alginate systems (Wang et al., 2021). A third strategy aims to engineer the scaffold’s interfacial properties to actively enhance cellular responsiveness. In this approach, the composite surface itself is designed to potentiate stem cell differentiation. A representative example features a biphasic calcium phosphate (BCP) scaffold co-doped with Sr^2+^, Mg^2+^, and Zn^2+^ to establish a cytocompatible, bioactive substrate, which is subsequently coated with poly (ester urea) (PEU). The synergistic combination of the ion-doped substrate and the PEU coating created a specific interface that significantly amplified the osteogenic differentiation of hUCMSCs under inductive conditions, demonstrating that the strategic integration of ionic cues with polymeric surface engineering can directly instruct stem cell fate through interfacial modulation (Pereira et al., 2023). These functionalized composites effectively direct hUCMSCs fate via specific bioactive cues, as exemplified by the activation of the Wnt/β-catenin pathway by EGCG, thereby enhancing bone regeneration *in vivo* (Wang et al., 2021).

Similarly, to address the critical need for vascularization in bone regeneration, composites have been designed to deliver pro-angiogenic cues. A potent approach is the strategic doping of scaffolds with bioactive ions. For example, copper (Cu)-doped nHA/CS/gelatin composites leverage released Cu ions to stabilize hypoxia-inducible factor 1-alpha (HIF-1α), thereby upregulating VEGF and concurrently enhancing osteogenesis through improved collagen maturation (Bozorgi et al., 2024). Alternatively, composites can be engineered to incorporate natural reservoirs of growth factors (e.g., human umbilical cord serum) (Jang et al., 2021) or specific pro-angiogenic molecules (e.g., the flavonoid naringin) (Zhao et al., 2021). These designs create multifunctional microenvironments that not only support stem cell osteogenesis but also enhance endothelial network formation, as evidenced by increased *in vitro* tube formation and *in vivo* bone healing.

Collectively, these functionalization strategies exemplify the evolution of composite scaffolds from passive, structurally optimized carriers to active, instruction-giving platforms. By deliberately embedding specific chemical, molecular, or ionic cues within the composite architecture, researchers can create sophisticated microenvironments that synergize with the inherent regenerative potential of hUCMSCs. This approach represents a promising strategy for future applications in reconstructing complex oral and maxillofacial defects, pending broader validation in relevant preclinical models.

## 4 Applications of hUCMSC-based scaffolds in oral and maxillofacial tissue engineering

The following sections detail the applications of hUCMSC-based scaffold strategies for regenerating bone/cartilage, periodontal, and pulp-dentin tissues. A summary of key representative studies in these areas is provided in Table 2.

**TABLE 2:** Representative preclinical and clinical studies utilizing hUCMSC-based scaffold strategies for oral and maxillofacial tissue regeneration.

​	Cell or exosome source	Scaffold materials (type)	Refs	Research model	Key outcome/Advantage
Bone	hUCMSCs	RGD-grafted CPC (Bioceramic)	(Chen et al., 2018)	Rat, critical-sized calvarial defect	Promoted osteogenesis and angiogenesis, comparable to hBMSCs
	hUCMSCs	RGD-grafted CPC (Bioceramic)	(Wang P. et al., 2015)	Rat, critical-sized calvarial defect	Promoted osteogenesis and angiogenesis, comparable to hBMSCs
	hUCMSCs	PRP (Biologically derived scaffold)	(Wen et al., 2014)	Rat, critical-sized calvarial defect	Enhanced bone regeneration; PRP maintained/promoted hUCMSC differentiation and osteogenic capacity
	hUCMSCs	Collagen gel (Polymer)	(Wen et al., 2013)	Rat, critical-sized calvarial defect	Enhanced bone healing
	WJMSCs	Bio-Oss® Collagen (Composite)	(Kosinski et al., 2020)	Rat, critical-sized calvarial defect	Synergistic effect enhanced bone healing
	hUCMSCs	nHA/CS/PLGA scaffold (Composite)	(Wang F. et al., 2015)	Rat, critical-sized calvarial defect	Scaffold promoted osteogenic differentiation and enhanced defect repair
	WJMSCs	Cu-nHA/Cs/Gel scaffold (Composite)	(Bozorgi et al., 2024)	Rat, critical-sized calvarial defect	Cu doping promoted osteogenesis and angiogenesis, enhancing bone regeneration
	hUCMSCs	Gelatin (Polymer)	(Hendrijantini et al., 2018)	Rat, osteoporotic mandible defect	Promoted regeneration; upregulated TGF-β1/Runx2 and increased osteoblasts
	hUCMSCs	Bone collagen matrix (Biologically derived scaffold)	(Sun X. C. et al., 2023)	Rabbit, alveolar cleft model	Repaired defects and promoted bone formation; enhanced osteoblast, collagen I, saccharide and BMP-2 production; modulated proliferation/apoptosis
	hUCMSCs + BMP-2	HA/CS/Gel scaffold (Composite)	(Bangun et al., 2021)	Goat, alveolar cleft defect model	Achieved regeneration comparable to autograft
	UCMSCs (Autologous)	PLGA nanomicrofiber scaffold (Polymer)	(Caballero et al., 2017)	Juvenile swine, alveolar cleft model	Autologous cell-scaffold combo showed promise for repair
	hUCMSCs	Gelatin (Polymer)	(Prasetyo et al., 2024)	Diabetic rat, apical periodontitis model	Mitigated periapical inflammation and promoted angiogenesis (M1-to-M2 shift)
	hUCMSCs	Gelatin (Polymer)	(Prasetyo et al., 2025)	Diabetic rat, apical periodontitis model	Increased osteoblasts, enhanced bone formation/maturation via Runx2/Osterix
​	WJMSCs	Collagen-nHA with Dex (Composite)	(Tanideh et al., 2025)	Rat, mandibular defect model	Maximized osteoblast/osteocyte increase and mandibular bone regeneration (synergy with Dex)
	hUCMSCs + PRP	3D-printed PCL/TCP honeycomb scaffold (Composite)	(Nam et al., 2023)	Minipig, sinus augmentation and implantation model	Enhanced bone formation and implant osseointegration; scaffold offered structural support/controlled degradation
	hUCMSCs	Gelatin (Polymer)	(Kuntjoro et al., 2023)	Diabetic rat, femoral implant model	Enhanced osseointegration in diabetes
	hUCMSCs (Pre-differentiated, osteogenic)	Polydopamine-coated SMP scaffold based on cross-linked PCL (Composite)	(Guda et al., 2024)	Rat, critical-sized calvarial defect	Pre-differentiated hUCMSCs outperformed undifferentiated hUCMSCs, BMSCs and ADSCs in bone formation
	hUCMSCs (Co-culture: Osteogenic + Angiogenic)	3D-printed β-TCP (Bioceramic)	(Rong et al., 2020)	Rat, critical-sized calvarial defect	Optimized co-culture (3:1) yielded superior bone repair vs. osteogenic cells alone
	hUCMSCs (Genetically engineered: overexpressing circCTTN)	GelMA hydrogel (Polymer)	(Zhang M. et al., 2023)	Rat, critical-sized calvarial defect	Achieved bone repair and established the critical osteogenic role of circCTTN.
	hUCMSC-Exos	Bio-Gide collagen membrane (Biologically derived scaffold)	(Li H. et al., 2025)	Aged rat, critical-sized cranial defect	Promoted bone regeneration in aged rats by mitigating cellular senescence via autophagy restoration
	hUCMSC-Exos	PVA hydrogel (Polymer)	(He et al., 2024)	Rat, alveolar socket defect	Outperformed free exosomes in enhancing bone regeneration, primarily mediated by exosomal miR-21-5p
	hUCMSC-Exos	CS/HA scaffold (Composite)	(Bahar et al., 2022)	Rat, critical-sized cranial defect	Synergistic tri-component construct promoted regeneration of critical-sized defects, outperforming single/dual components
	hUCMSC-Exos	3D-printed SF/COL-I/nHA scaffold (Composite)	(Sun X. et al., 2023)	Rat, alveolar bone defect model	Enabled sustained exosome release, promoting bone regeneration and angiogenesis
	hUCMSC-Exos	HA hydrogel infiltrated 3D-printed nHP scaffold (Composite)	(Zhang et al., 2021)	Rat, critical-sized cranial defect	Sustained-release system promoted bone regeneration
	hUCMSC-Exos (OM-Exos, from osteogenic medium and PM-Exos, from proliferation medium)	3D-printed TCP scaffold (Bioceramic)	(Li S. et al., 2024)	Rat, critical-sized calvarial defect	OM-Exos outperformed PM-Exos in promoting hBMSC osteogenic differentiation and bone regeneration
Cartilage	hUCMSCs	PRF (Biologically derived scaffold)	(Sumarta et al., 2025)	Rabbit, mandibular condylar cartilage defect	Achieved superior fibrocartilage regeneration and enhanced biomechanical properties
Periodontal Tissue	hUCMSCs	β-TCP (Bioceramic)	(Shang et al., 2017)	Rat, inflammatory periodontal defect model	Promoted hard tissue regeneration and new functional PDL attachment under inflammation
	hUCMSCs (Osteogenically induced)	Porcine fibrin sealant gel (Biologically derived scaffold)	(Li et al., 2014)	Mouse, subcutaneous implantation (dentin disc)	Osteogenic induction enhanced cementum-like tissue formation with PDL-like fiber attachment
	hUCMSCs	PCL scaffold (Polymer)	(Kadam et al., 2019)	Human (clinical case), Miller’s Class II recession	Achieved >80% root coverage, demonstrating clinical efficacy
	hUCMSC-Exos	PVA/sodium alginate hydrogel (Polymer)	(Li K. et al., 2025)	Mouse, ligation-induced periodontitis model	Attenuated inflammation and inhibited bone resorption via exosomal miRNAs
Pulp-Dentin	WJMSCs	Decellularized human dental pulp (Biologically derived scaffold)	(Tan et al., 2024)	Feline, mature canine teeth (pulpectomy model)	Formed pulp-like tissues in root canals
	hUCMSCs	CGF (Biologically derived scaffold)	(Wang et al., 2024)	Bama miniature pig, dental caries model	Promoted necrotic pulp repair and formed organized odontoblast layer with predentin
	hUCMSCs	Matrigel-derived hydrogel (Biologically derived scaffold)	(Sabeti et al., 2024)	Dog, immature permanent teeth with irreversible pulpitis	Facilitated pulp-dentin complex regeneration without inflammation (vital apex required)
	hUCMSCs	PPP (Biologically derived scaffold)	(Angelopoulos et al., 2022)	Mouse, subcutaneous implantation (human dentin disc)	Enhanced hemoglobin content, angiogenesis and new dentin deposition
	hUCMSCs (Co-culture: V-hUCMSCs + hUCMSCs)	I collagen scaffolds (Polymer)	(Zhang et al., 2020a)	Mouse, subcutaneous implantation (human root segment)	Enhanced vascularization and pulp regeneration via HIF1A-AS2/HIF-1 pathway
	hUCMSCs (Co-culture: hUCMSCs + HUVECs)	I collagen scaffolds (Polymer)	(Zhang et al., 2020b)	Mouse, subcutaneous implantation (human root segment)	Enhanced vascularization and pulp regeneration via HIF1A-AS2/HIF-1 pathway
	hUCMSCs	PPP (Biologically derived scaffold)	(Brizuela et al., 2024)	Human (clinical case), pulp necrosis and periapical periodontitis	Achieved pulp revitalization and satisfactory healing (5-year follow-up)
	hUCMSCs	PPP (Biologically derived scaffold)	(Brizuela et al., 2020)	Human (RCT), regenerative endodontic therapy	Achieved clinical success in all patients (12-month)
​	hUCMSCs	PPP (Biologically derived scaffold)	(Cordero et al., 2020)	Human (clinical case), apical periodontitis with root perforation	Feasible for complex cases; induced periapical lesion regression and dentin bridge formation
	hUCMSC-Exos	CS (Polymer)	(Jafari et al., 2025)	Human (clinical case), irreversible pulpitis	Provided preliminary clinical evidence for stable healing, periapical repair and tissue integration

### 4.1 Bone and cartilage regeneration

This section discusses the application of hUCMSC-based strategies for both bone and cartilage regeneration in the oral and maxillofacial region. The key strategies, including cell and exosome-based approaches, optimization methods, regenerative mechanisms, and target tissues for both bone and cartilage are schematically summarized in Figure 4.

**FIGURE 4:**
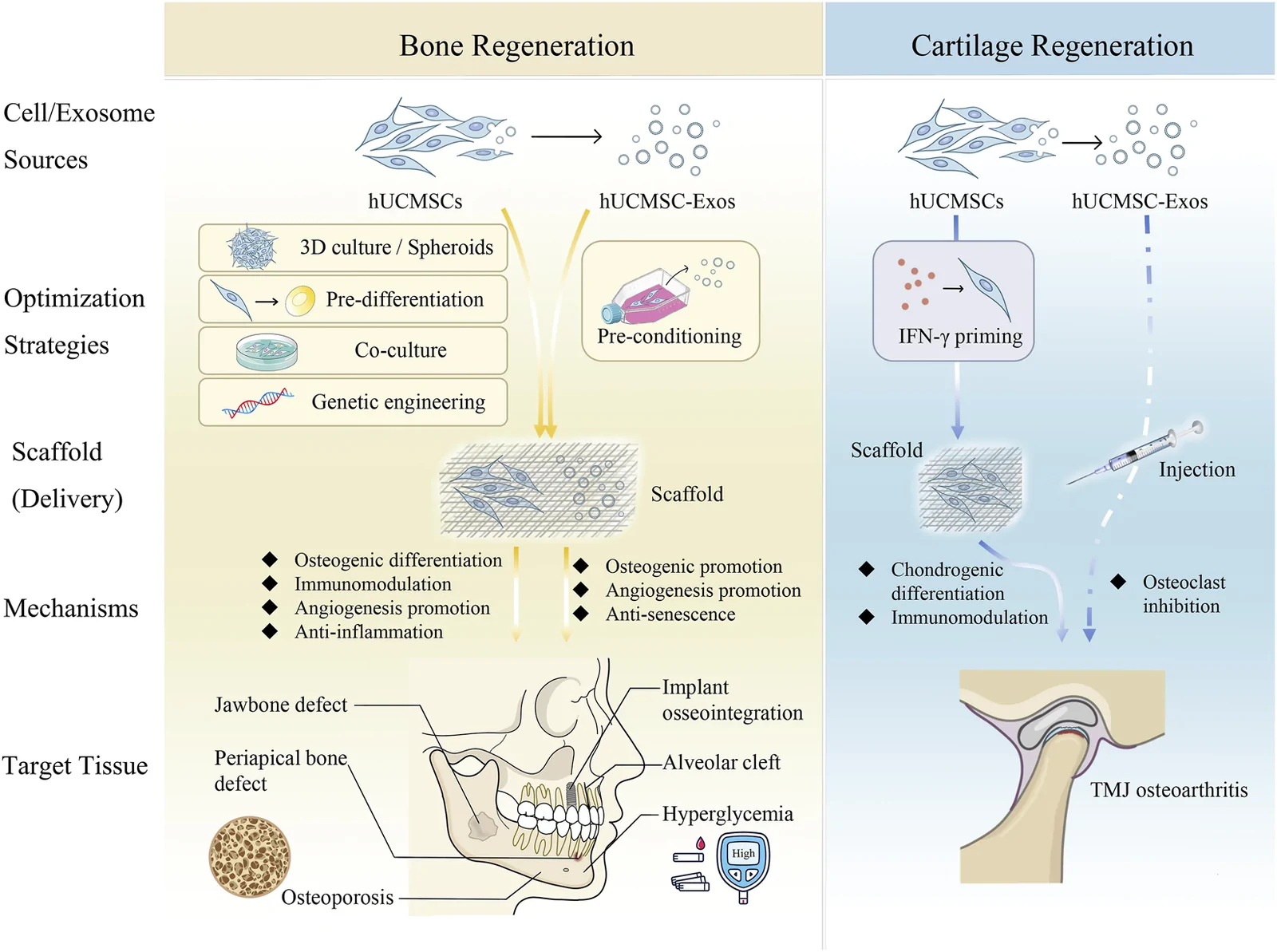
hUCMSC-Based Strategies for Oral and Maxillofacial Bone and Cartilage Regeneration. The yellow background denotes bone regeneration, and the blue background denotes cartilage regeneration. The figure is organized top-to-bottom to illustrate cell/exosome optimization strategies, regenerative mechanisms, and target tissues. Solid arrows indicate scaffold-based strategies with established evidence; dotted arrows indicate injection-based studies that provide a foundation for future scaffold-based delivery systems. Abbreviations: hUCMSCs (human umbilical cord mesenchymal stem cells); hUCMSC-Exos (hUCMSC-derived exosomes); 3D (three-dimensional); IFN-γ (interferon-γ); TMJ (temporomandibular joint).

#### 4.1.1 Scaffold-based delivery of hUCMSCs for bone regeneration

hUCMSCs represent a promising candidate for bone tissue regeneration. Their regenerative mechanisms are multifaceted, involving direct osteogenic differentiation and indirect stimulation of osteoblast and osteoclast maturation (Kosinski et al., 2020). Notably, their osteogenic capacity is well-established and comparable to that of hBMSCs (Chen et al., 2018; Wang P et al., 2015). Furthermore, hUCMSCs promote angiogenesis, primarily by stimulating VEGF production (Kosinski et al., 2020; Wang P et al., 2015). Critically, hUCMSCs exhibit potent immunomodulatory effects. These include suppressing inflammasome activation in macrophages, instructing macrophage polarization towards an anti-inflammatory M2 phenotype, and enhancing M2 macrophage recruitment to sites of injury (Deng et al., 2020). Concurrently, they elevate anti-inflammatory cytokine expression while inhibiting pro-inflammatory responses (Hendrijantini et al., 2023). This immunomodulatory capacity alleviates chronic inflammation, thereby creating a favorable microenvironment for bone regeneration. Within oral and maxillofacial surgery, these properties make hUCMSCs particularly suitable for applications such as jawbone defect repair and enhancing osseointegration around dental implants.

##### 4.1.1.1 Application of different scaffold materials

The critical-size calvarial defect model in rats serves as a standard platform for initial validation of hUCMSC-scaffold constructs for bone regeneration. Using this model, numerous studies have evaluated hUCMSCs with various scaffold categories—ranging from bioceramics and biologically derived scaffolds to polymers and composites. Among bioceramics, Chen et al. demonstrated that macroporous RGD-grafted CPC scaffolds significantly enhanced new bone and blood vessel formation (Chen et al., 2013; 2018), a finding later corroborated by Wang et al. (Wang P. et al., 2015). Biologically derived scaffolds also show promise: Wen et al. reported that PRP combined with hUCMSCs enhanced bone regeneration by maintaining hUCMSC differentiation and promoting their osteogenic capacity (Wen et al., 2014). In the application of polymers, the same group showed that collagen gel with hUCMSCs significantly improved bone healing (Wen et al., 2013).

Composite materials, designed to synergize the advantages of individual components, have been a major focus and demonstrate the potential of engineered microenvironments. Kosinski et al. implanted WJMSCs with Bio-Oss® Collagen into rat calvarial defects, observing a synergistic enhancement in bone healing (Kosinski et al., 2020). In another approach, Wang et al. developed an nHA/CS/PLGA scaffold. They demonstrated that this scaffold exhibits excellent physical properties and osteoinductive capacity, effectively promoting the osteogenic differentiation of hUCMSCs (Wang et al., 2014). Subsequent *in vivo* studies confirmed its efficacy in repairing rat critical-sized calvarial defects (Wang F. et al., 2015). Further investigating this scaffold, Su et al. found it sustains hUCMSC stemness by activating a pro-regenerative gene network that encompasses the BMP pathway, basic fibroblast growth factor (bFGF), VEGF, and C-X-C motif chemokine ligand 5 (CXCL5), which collectively support cell proliferation, differentiation, migration, and angiogenesis (Su et al., 2020). This work underscores the scaffold’s capacity to preserve the functional potency of hUCMSCs, a critical advantage for clinical translation. Similarly, Bozorgi et al. employed a copper-doped nHA/CS/gelatin scaffold with fibrin-encapsulated WJMSCs to promote osteogenesis and angiogenesis in a rat calvarial defect model (Bozorgi et al., 2024). These studies collectively illustrate that composite scaffold design is not merely about combining materials, but about creating a synergistic microenvironment that can maintain stem cell vitality and instruct their fate.

Beyond the standardized calvarial defect model, the therapeutic potential of hUCMSC-scaffold systems has been further investigated in more clinically relevant and challenging scenarios that closely mimic human pathologies. These include addressing regeneration under compromised systemic conditions (e.g., osteoporosis, diabetes), repairing anatomically and functionally complex defects (e.g., alveolar cleft, periapical lesions), enhancing osseointegration in implant dentistry, and achieving functional restoration in critical-sized defects of load-bearing jawbones (e.g., mandibular defects). This expansion beyond the ideal calvarial model underscores the adaptability and therapeutic promise of hUCMSC-scaffold systems in addressing the multifaceted challenges of real-world maxillofacial repair.

As a representative systemic compromise, osteoporosis disrupts jawbone metabolism and reduces bone mass, leading to defects characterized by poor quality and delayed healing. The repair of these osteoporotic jaw defects, therefore, presents a considerable clinical challenge (Hong et al., 2025). Hendrijantini et al. employed gelatin solvent as a carrier to deliver hUCMSCs into the mandibles of osteoporotic rats. The treatment demonstrated a significant regenerative effect on the osteoporotic bone. Mechanistically, this outcome was attributed to the upregulation of TGF-β1 and Runt-related transcription factor 2 (Runx2), alongside an increase in osteoblast levels (Hendrijantini et al., 2018).

The repair and reconstruction of alveolar clefts, as a common type of maxillofacial bone defect, has long been a key focus of research. In a rabbit alveolar cleft model, Sun et al. showed that a bone collagen matrix with hUCMSCs effectively repaired bone defects, promoting osteoblast production, type I collagen deposition, BMP-2 expression, and new bone formation, while also modulating proliferation and apoptosis-related processes (Sun X. C. et al., 2023). In a goat alveolar cleft model, Bangun et al. demonstrated that an HA/CS/gelatin scaffold carrying both BMP-2 and hUCMSCs achieved bone regeneration comparable to autografts, with more efficient surgery and faster recovery (Bangun et al., 2021). Similarly, Caballero et al. investigated tissue engineering strategies for congenital alveolar cleft repair using a juvenile swine surgical model. They fabricated nanomicrofiber scaffolds (NMFS) from resorbable PLGA by electrospinning, and seeded autologous UCMSCs—harvested from the same swine at birth—onto the scaffolds. The results showed that this autologous cell-scaffold combination effectively repaired alveolar clefts, representing a promising candidate for clinical translation (Caballero et al., 2017; Caballero et al., 2015).

Apical periodontitis (AP) causes periapical bone resorption, exacerbated by hyperglycemia-induced chronic inflammation (Prasetyo et al., 2024). In a diabetic rat AP model, hUCMSCs delivered in a gelatin scaffold mitigated inflammation and promoted angiogenesis, favorably remodeling the periapical niche (Prasetyo et al., 2024). A follow-up study using the same model showed increased osteoblast numbers and enhanced bone formation and maturation (Prasetyo et al., 2025). Together, these studies delineate a coherent two-phase therapeutic mechanism: hUCMSCs first establish a conducive regenerative microenvironment by dampening inflammation and enhancing vascularization through a shift in macrophage polarization from the M1 (pro-inflammatory) to the M2 (pro-reparative) phenotype. This microenvironment then permits and actively drives the subsequent osteogenic differentiation program (mediated by Runx2/Osterix activation), which is necessary for structural bone repair (Prasetyo et al., 2024; Prasetyo et al., 2025). These findings highlight the therapeutic potential of hUCMSC-gelatin constructs for diabetic periapical bone defects.

Mandibular reconstruction following trauma, tumor resection, or severe atrophy is challenging due to its load-bearing and aesthetic functions (Fatani et al., 2022). Scaffold-based hUCMSC strategies offer promise for critical-sized mandibular defects. For instance, Tanideh et al. used a porous collagen-nHA scaffold loaded with dexamethasone (Dex) and WJMSCs in a rat model, achieving enhanced osteoblast/osteocyte numbers and mandibular bone regeneration. This demonstrates the potential of scaffold-cell-inductive factor combinations for mandibular reconstruction (Tanideh et al., 2025).

In terms of osseointegration of implants, Nam et al. developed a 3D-printed PCL/TCP honeycomb scaffold and evaluated it in a minipig sinus augmentation and dental implantation model. The scaffold provided structural support, promoted cell adhesion and vascular infiltration, and degraded at a controlled rate to avoid acidosis. This system enhanced bone formation and osseointegration, with hUCMSCs serving as the osteogenic source and PRP providing bioactive factors (Nam et al., 2023). Moreover, diabetes is a significant factor affecting the success of osseointegration in dental implants. Kuntjoro et al. delivered hUCMSCs in gelatin around titanium implants in diabetic rat femurs, showing enhanced osseointegration under diabetic conditions (Kuntjoro et al., 2023). These findings support the therapeutic potential of hUCMSCs for implant osseointegration.

##### 4.1.1.2 Optimization of cellular strategies

Beyond the selection of scaffolds, strategies centered on preconditioning or engineering the cells themselves have gained prominence. By modulating cell morphology, priming differentiation, or editing key genes, researchers can actively “program” hUCMSCs to better overcome the challenges of survival, vascularization, and ultimately, bone formation in complex defects.

Physical pretreatment, such as forming hUCMSCs into three-dimensional (3D) spheroids, represents a direct strategy to enhance their therapeutic function. This 3D configuration strengthens cell–ECM interactions, a mechanism known to sustain pluripotency and promote osteogenesis (Wei et al., 2025). Wei et al. demonstrated that spontaneously formed hUCMSC spheroids, when combined with β-TCP scaffolds, significantly upregulated osteogenic gene expression and enhanced differentiation and bone regeneration compared to conventional two-dimensional (2D) monolayer cultures (Wei et al., 2025).

Directing hUCMSCs toward an osteogenic lineage through pre-differentiation prior to implantation is another potent strategy to boost bone formation. This approach ensures that a pre-committed osteogenic cell population is delivered, potentially accelerating the regenerative process (Xu et al., 2019). Strong support comes from a comparative study using a polydopamine-coated SMP scaffold to evaluate the bone-forming capacity of pre-differentiated versus undifferentiated MSCs from different sources. Pre-differentiated hUCMSCs generated significantly more new bone than their undifferentiated counterparts or pre-differentiated MSCs from bone marrow or adipose tissue in a critical-sized calvarial defect model. This comparison supports osteogenic pre-differentiation as a key strategy for enhancing hUCMSC bone regeneration (Guda et al., 2024).

Given the synergistic effect of osteogenic and angiogenic cells in co-culture systems, defining the optimal cellular composition is a key challenge. Using a homologous cell source on 3D-printed β-TCP scaffolds, Rong et al. found that a 3:1 ratio of osteogenic to angiogenic hUCMSCs yielded superior bone repair to osteogenic cells alone (4:0) in rat calvarial defects. This study confirms the importance of precise ratio optimization and highlights the need for standardized pre-induction and co-culture parameters for clinical translation (Rong et al., 2020).

Beyond physical pretreatment and co-culture strategies, genetic engineering offers a more precise and fundamental approach to enhance hUCMSC function. For instance, Zhang et al. used gelatin-methacrylate (GelMA) hydrogels to deliver hUCMSCs overexpressing circCTTN in rat calvarial defects, achieving effective bone repair and establishing circCTTN’s role in driving osteogenic differentiation. This suggests that genetic editing can enhance hUCMSC therapeutic efficacy in bone tissue engineering (Zhang M. et al., 2023).

#### 4.1.2 Exosome-functionalized scaffolds for bone regeneration

As previously established, the paracrine effect serves as a key mechanism through which hUCMSCs exert their reparative functions, with exosomes representing the core mediators in this process. Based on this rationale, loading hUCMSC-derived exosomes (hUCMSC-Exos) onto biological scaffolds constitutes a transformative cell-free paradigm in bone tissue engineering. To unlock the full therapeutic potential of this strategy, research has converged on two synergistic frontiers: the engineering of advanced scaffolds for optimized exosome delivery and bioactivity, and the directed enhancement of the exosomes themselves (Villani et al., 2024).

The optimization of exosome delivery scaffolds follows a spectrum of strategies, ranging from leveraging existing clinical materials to pioneering novel designs. These approaches can be conceptually categorized by their translational immediacy and design complexity. A prevalent strategy is the use of commercially available materials—for instance, Li et al. adsorbed hUCMSC-Exos onto Bio-Gide, a native collagen membrane, and showed effective bone regeneration in aged rat cranial defects. Mechanistically, the exosomes restored autophagy in jaw bone-marrow mesenchymal stem cells (JBMMSCs) via PI3K/AKT/mTOR (phosphatidylinositol 3-kinase/protein kinase B/mammalian Target of Rapamycin) modulation, mitigating cellular senescence and rejuvenating the endogenous stem cell pool (Li H. et al., 2025). This anti-aging mechanism is particularly relevant for regeneration in aged patients.

Formulation engineering with well-established biomaterials is another versatile approach. He et al. encapsulated hUCMSC-Exos in a polyvinyl alcohol (PVA) hydrogel (Exo@PVA), which enhanced bone regeneration in rat alveolar sockets more effectively than free exosomes, improving BMSC and human umbilical vein endothelial cells (HUVECs) osteogenic differentiation. Mechanistically, exosomal miR-21-5p promoted osteogenesis by inhibiting WWP1 (E3 ubiquitin ligase WWP1)-mediated KLF5 ubiquitination and enhanced angiogenesis by targeting ATP2B4 (also known as PMCA4) (He et al., 2024), confirming that the delivery scaffold is essential for maximizing exosome therapeutic potential.

At the frontier of scaffold design lie innovative platforms conceived and fabricated *de novo* to integrate structural, mechanical, and biochemical cues. For instance, Bahar et al. showed that CS/HA scaffolds loaded with hUCMSC-Exos synergistically repaired critical-sized cranial defects in rats, outperforming single-component scaffolds (Bahar et al., 2022). Sun et al. developed a low-temperature 3D-printed silk fibroin, collagen I, and nHA (SF/COL-I/nHA) scaffold that enabled sustained exosome release and promoted bone regeneration and angiogenesis in alveolar defects, with released exosomes enhancing HUVEC proliferation, migration, and tube formation (Sun X. et al., 2023). Zhang et al. designed an injectable hyaluronic acid hydrogel-infiltrated 3D-printed nHP scaffold for sustained exosome delivery, where exosomal miR-21 promoted angiogenesis via NOTCH1/DLL4 (notch receptor 1/delta-like ligand 4) inhibition and VEGFA (Vascular Endothelial Growth Factor A)/HIF-1α upregulation, indirectly facilitating osteogenesis without direct osteocyte induction (Zhang et al., 2021). Collectively, these studies exemplify the shift from passive carriers to actively instructive scaffolds that provide both mechanical and biochemical cues to maximize the regenerative potential of therapeutic exosomes.

Beyond optimizing the delivery vehicle, enhancing exosome potency through preconditioning of parent hUCMSCs—by modifying culture conditions to alter exosome composition and function—is another key strategy (Ma et al., 2023). For instance, Li et al. integrated exosomes from osteogenically preconditioned hUCMSCs (OM-Exos) into 3D-printed TCP scaffolds and found that they promoted superior osteogenic differentiation and bone regeneration compared to exosomes from non-preconditioned cells (PM-Exos), an effect likely attributable to differences in miRNA profiles (Li S. et al., 2024). This highlights preconditioning as a potent approach to tailor exosome therapeutic profiles for specific applications.

In these studies, hUCMSC-Exos were loaded either by passive adsorption onto scaffold surfaces or by physical encapsulation within hydrogel or porous matrices (He et al., 2024; Li H. et al., 2025). For encapsulated systems, release has been reported to be governed by scaffold degradation and diffusion (Zhang et al., 2021).

#### 4.1.3 Cartilage regeneration applications

For cartilage repair, hUCMSCs exert their therapeutic effects through a dual mechanism: direct chondrogenic differentiation to replenish cartilage tissue (Fu et al., 2023), and potent paracrine signaling to modulate inflammation and immune responses (Perry et al., 2020; Zhang P. et al., 2023). In oral and maxillofacial surgery, these strategies are primarily applied to regenerate cartilage and repair the TMJ.

Direct intra-articular injection of hUCMSCs has demonstrated therapeutic efficacy in preclinical TMJ osteoarthritis (TMJOA) models, validating their potential. For example, one study demonstrated that in a rabbit model of TMJOA, intra-articular injection of hUCMSCs promoted cartilage regeneration and exerted anti-inflammatory effects (Kim et al., 2019). In a separate study utilizing a rat TMJOA model, Kim et al. further demonstrated that the therapeutic potency could be enhanced through cell priming; specifically, injection of interferon-γ (IFN-γ)-stimulated hUCMSCs significantly alleviated pain and facilitated tissue repair by potently polarizing synovial macrophages toward the anti-inflammatory M2 phenotype (Kim Y. et al., 2025).

Despite promising outcomes, injection-based delivery faces inherent challenges, including poor cell retention, lack of structural support, and the need for repeated administrations (Ma et al., 2026). Consequently, research has shifted toward combining hUCMSCs with 3D bioactive scaffolds to provide a supportive niche for enhanced cell survival, organized tissue growth, and sustained bioactive factor release (Baecher et al., 2025). *In vitro*, Ramzan et al. showed that dUC-derived hydrogel supported hUCMSC chondrogenic differentiation, suggesting potential for cartilage-like tissue engineering (Ramzan et al., 2022). *In vivo*, Sumarta et al. demonstrated that hUCMSCs combined with a PRF scaffold achieved superior repair of mandibular condylar cartilage defects in rabbits, via hUCMSC fibrochondrogenic differentiation and ECM synthesis (glycosaminoglycans, collagens I and II), alongside PRF’s sustained growth factor release (Sumarta et al., 2025). These findings support the promise of hUCMSC-PRF constructs for TMJ cartilage regeneration.

As noted previously, hUCMSCs primarily mediate joint injury repair through paracrine mechanisms (Perry et al., 2020). Research on hUCMSC-Exos has preliminarily validated their therapeutic potential and mechanisms in TMJ cartilage regeneration. For instance, Yang et al. demonstrated that intra-articular injection of hUCMSC-Exos (referred to in the study as hUCMSC-EVs) protects cartilage and remodels subchondral bone in a rat model of TMJOA. Mechanistically, these vesicles deliver let-7a-5p, which inhibits osteoclast differentiation and mitigates subchondral bone loss by targeting integrin β3 (ITGβ3), thereby promoting cartilage matrix homeostasis. These findings suggest hUCMSC-Exos are a promising early intervention for TMJOA (Yang et al., 2025).

However, similar to cell-based therapies, the clinical translation of exosome therapy is constrained by the limitations of direct injection. To date, studies combining hUCMSC-Exos with scaffolds for tissue regeneration in the TMJ are notably lacking. Therefore, designing advanced scaffold systems capable of immobilizing, protecting, and providing sustained release of hUCMSC-Exos represents a critical path forward for overcoming current bottlenecks and achieving functional TMJ regeneration (Ma et al., 2026).

### 4.2 Periodontal tissue regeneration

Periodontal disease is a common disorder that can cause irreversible damage to the tooth-supporting tissues, including the cementum, periodontal ligament, and alveolar bone, ultimately resulting in tooth loss. The ultimate goal of periodontal therapy is the functional regeneration of this complex tissue architecture (Shaikh et al., 2022; Sheehy et al., 2026). Advances in tissue engineering have positioned cell-based strategies, particularly those employing hUCMSCs, at the forefront of this endeavor. The rationale for using hUCMSCs is well-founded: they possess the capacity to differentiate into periodontal lineage cells, such as human periodontal ligament cells (hPDLCs) (Heinayati et al., 2025). Moreover, it has been demonstrated that hUCMSCs can differentiate into cells capable of adhering to root surfaces, which exhibit a fibroblast-like morphology, and the supplementation of basic fibroblast growth factor (bFGF) further enhances the proliferation, attachment, and maturation of these cells (George et al., 2015). Importantly, under chronic inflammatory conditions typical of periodontitis, hUCMSCs display stronger anti-inflammatory properties compared to endogenous human periodontal ligament stem cells (hPDLSCs), rendering them particularly promising for periodontal regeneration (Shang et al., 2017). Together, these findings underscore the considerable potential of hUCMSCs for application in periodontal tissue regeneration (Figure 5).

**FIGURE 5:**
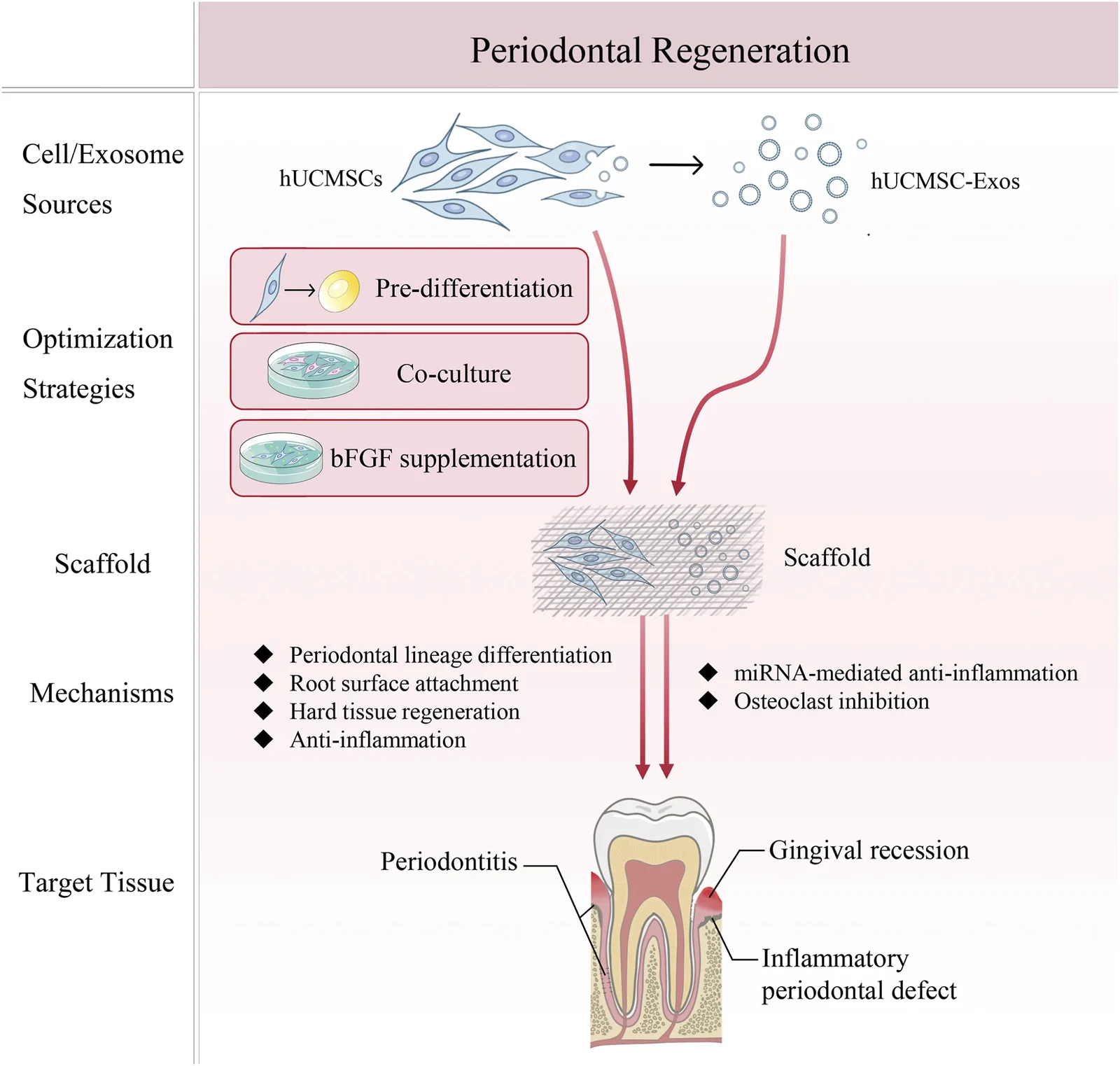
hUCMSC-Based Strategies for Periodontal Regeneration. The figure is organized top-to-bottom to illustrate cell/exosome sources, optimization strategies, scaffold delivery, regenerative mechanisms, and target tissues. Abbreviations: hUCMSCs (human umbilical cord mesenchymal stem cells); hUCMSC-Exos (hUCMSC-derived exosomes); bFGF (basic fibroblast growth factor); miRNA (microRNA).

#### 4.2.1 Scaffold-based delivery of hUCMSCs for periodontal tissue regeneration

One of the key challenges in applying hUCMSCs for periodontal tissue regeneration lies in their precise delivery and retention at the complex, three-dimensional tooth root surface. To overcome this, biomaterial scaffolds have been engineered not merely as carriers, but as bioactive platforms that can localize cells and provide conducive signals for regeneration, with promising outcomes emerging from both preclinical and clinical studies (Sheehy et al., 2026).

CGF, known to promote favorable healing outcomes in tissue regeneration during periodontal surgical procedures (Lei et al., 2020), has been explored as a scaffold. Li et al. co-cultured hUCMSCs with human periodontal ligament fibroblasts (HPLFs) on CGF membranes, finding that this tri-culture system synergistically enhanced HPLF function. Mechanistically, this effect was linked to the upregulation of Tafazzin (TAZ) and osteogenic genes, highlighting the CGF membrane’s role in facilitating hUCMSC-mediated periodontal regeneration (Li et al., 2021).

Beyond *in vitro* evidence, the regenerative efficacy of hUCMSCs delivered via scaffolds has been compellingly demonstrated *in vivo*. Shang et al. established an inflammatory periodontal defect model in rats to evaluate the combined effect of hUCMSC aggregates and β-TCP on periodontal tissue regeneration. Their findings demonstrate that this combination promotes the regeneration of periodontal hard tissues under inflammatory conditions and facilitates the formation of new functional periodontal ligament attachments (Shang et al., 2017). To more specifically assess the potential of hUCMSCs to regenerate the cementum-periodontal ligament interface, Li et al. combined osteogenically induced hUCMSCs with a porcine fibrin sealant gel and applied the mixture to a dentin disc before implanting the construct into the subcutaneous tissue of mice to simulate periodontal tissue regeneration. The results demonstrated that, compared to non-induced hUCMSCs, the experimental group generated a new cementum-like layer *in vivo*. This layer was associated with a fiber-like tissue attachment, morphologically resembling the periodontal ligament structure. These findings indicate that pre-differentiation potentiates hUCMSCs for forming structurally relevant periodontal tissues (Li et al., 2014).

Notably, this strategy has entered early clinical translation. In a clinical study by Kadam et al., a combination of hUCMSCs and a PCL scaffold was used to treat a patient with multiple gingival recessions classified as Miller’s Class II. Six months post-surgery, a significant reduction in gingival recession was observed, with over 80% root coverage achieved (Kadam et al., 2019). This result adds supportive clinical evidence for the scaffold-based delivery of hUCMSCs in periodontal soft tissue reconstruction.

#### 4.2.2 Exosome-functionalized scaffolds for periodontal tissue regeneration

Similarly, the therapeutic potential of hUCMSC-Exos has emerged as a major cell-free strategy in periodontal regeneration. hUCMSC-Exos modulate multiple inflammatory pathways by regulating key cellular factors, thereby influencing cell metabolism and differentiation to promote tissue repair (Gao et al., 2024; Li K. et al., 2025). Fundamental *in vitro* studies substantiate this potential. For instance, hUCMSC-Exos have been shown to directly enhance the proliferation and osteogenic differentiation of hPDLSCs, with mechanistic insights pointing to the activation of key signaling pathways such as PI3K/AKT (Yang et al., 2022). This evidence underscores the inherent capacity of hUCMSC-Exos to stimulate the regeneration of periodontal tissues, a potential that may be particularly valuable in challenging pathological contexts, including diabetic periodontitis, where the native regenerative capacity is often impaired (Li K. et al., 2025).

To translate this intrinsic bioactivity into effective and localized therapeutic outcomes, strategies for exosome delivery and retention are essential. Li et al. developed an injectable hydrogel system (exo@H) for periodontal regeneration by cross-linking polyvinyl alcohol and sodium alginate to encapsulate lyophilized hUCMSC-Exos. In a mouse model of ligation-induced periodontitis, exo@H effectively attenuated periodontal inflammation and inhibited alveolar bone resorption. Mechanistic studies revealed that the therapeutic efficacy was mediated by the exosome-delivered miRNAs, particularly let-7f-5p and miR-203-3p, which target the anti-inflammatory factor interleukin-13 (IL-13) and nitrilase-like protein 2 (Nit2), respectively, to coordinately dampen inflammation and enhance tissue repair (Li K. et al., 2025). This study highlights the efficacy of a hydrogel-based delivery platform in augmenting the therapeutic potential of hUCMSC-Exos for periodontal regeneration.

### 4.3 Pulp-dentin complex regeneration

Deep caries and trauma can cause dentin defects and pulp injuries, which frequently lead to irreversible damage of the dentin-pulp complex and, ultimately, tooth necrosis (Kim and Kim, 2025). The prevailing clinical challenge lies in the limitations of current interventions, such as root canal treatment: while effective for infection control, they lack the capacity to induce true biological regeneration of this complex structure (Han et al., 2024). Consequently, achieving the simultaneous regeneration of functional, vascularized, and innervated pulp tissue alongside dentin matrix formation constitutes a central challenge in oral regenerative medicine. To address this, tissue engineering strategies employing hUCMSCs and scaffold systems present a promising approach, as they can provide both an inductive biomimetic microenvironment and a potent cellular source (Sheehy et al., 2026).

The odontogenic and multi-lineage potential of hUCMSCs forms the biological foundation for this approach. Studies have demonstrated that *in vitro* induction with tooth germ cell conditioned medium (TGC-CM) or liquid extract of human treated dentin matrix (LE-TDM) could promote hUCMSCs differentiation into odontoblast-like cells (Chen et al., 2015). Furthermore, Zhang et al. confirmed that under VEGF induction, hUCMSCs can differentiate into endothelial cell lineages. The capacity for dual differentiation into both odontogenic and vasculogenic lineages directly addresses the two fundamental components of the dentin-pulp complex: a mineralized matrix and a functional vascular network (Zhang et al., 2020a). Consequently, hUCMSCs are positioned as a uniquely versatile cellular source for holistic pulp-dentin regeneration (Figure 6).

**FIGURE 6:**
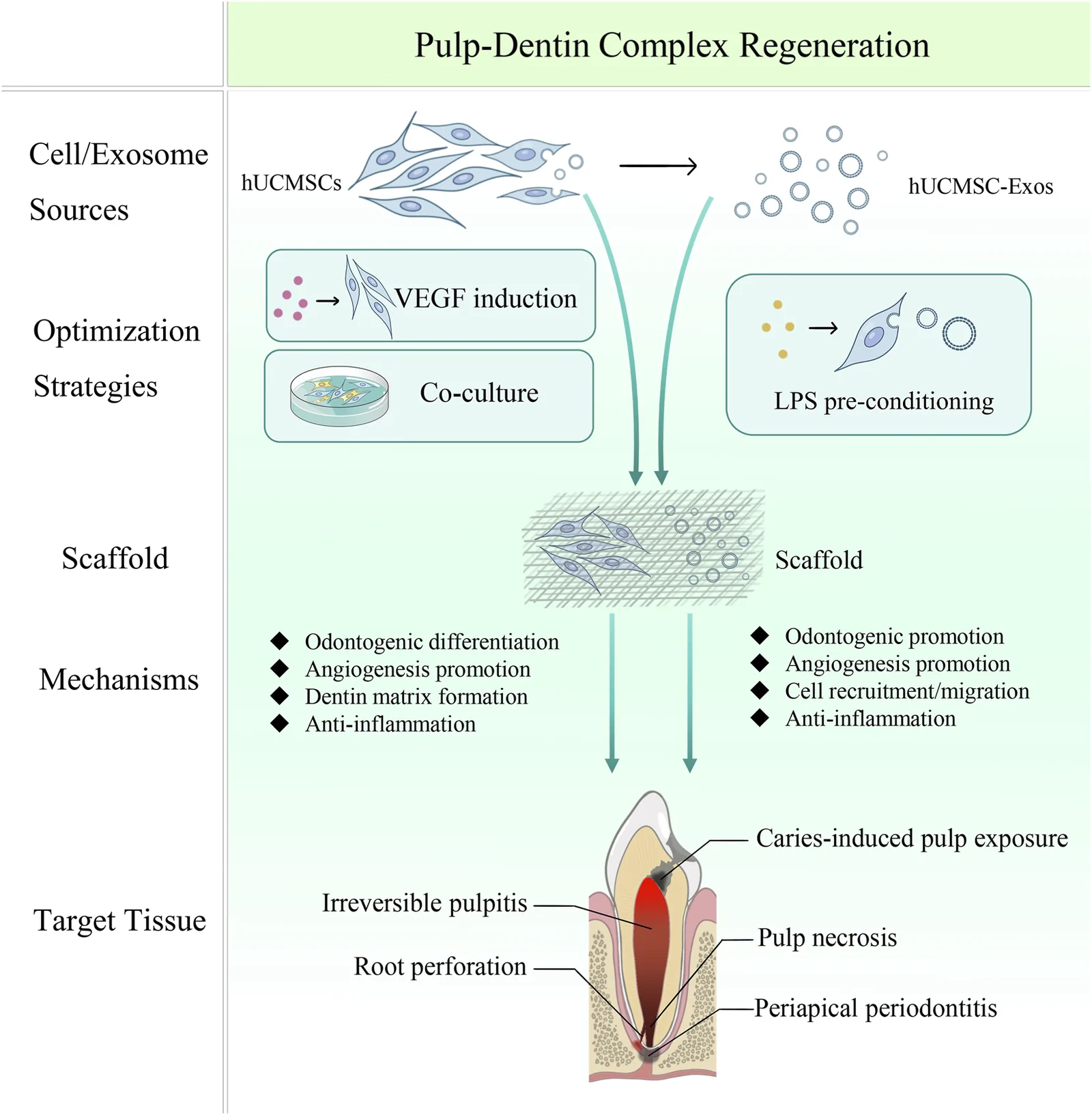
hUCMSC-Based Strategies for Pulp-Dentin Complex Regeneration. The figure is organized top-to-bottom to illustrate cell/exosome sources, optimization strategies, scaffold delivery, regenerative mechanisms, and target tissues. Abbreviations: hUCMSCs (human umbilical cord mesenchymal stem cells); hUCMSC-Exos (hUCMSC-derived exosomes); VEGF (vascular endothelial growth factor); LPS (lipopolysaccharide).

#### 4.3.1 Scaffold-based delivery of hUCMSCs for pulp-dentin regeneration

##### 4.3.1.1 Application of different scaffolds

Building on the aforementioned foundational potential, a number of studies have utilized animal models to validate the therapeutic prospects of combining hUCMSCs with various scaffold materials for dental pulp and dentin regeneration.

Tan et al. demonstrated pulp-like tissue formation using a decellularized human dental pulp (DHDP) scaffold combined with WJMSCs in a feline model of mature canine teeth. This work underscores the advantage of tissue-specific ECM scaffolds in providing native biochemical and structural cues for odontogenic differentiation, supporting their promise for functional pulp regeneration following pulpectomy (Tan et al., 2024). Beyond ECM-derived scaffolds, growth factor-rich autologous products such as CGF have also shown promise. In a Bama miniature pig caries model, Wang et al. combined hUCMSCs with a CGF scaffold to form engineered pulp-like tissue, with organized odontoblast layers, predentin deposition, and increased cell numbers (Wang et al., 2024). These findings support the efficacy of hUCMSC-CGF constructs and highlight their promise as growth factor-rich scaffolds for pulp regeneration in clinically relevant large-animal models.

Beyond demonstrating regenerative capacity, a critical advancement lies in the ability of hUCMSC-based strategies to actively resolve inflammation while promoting regeneration. This dual role was clearly elucidated by Sabeti et al., who evaluated the therapeutic potential of hUCMSCs combined with a Matrigel-derived hydrogel scaffold in immature dog permanent teeth exhibiting root canal inflammation. The *in vivo* findings suggest that hUCMSCs, when applied as part of a regenerative endodontic procedure, can facilitate the regeneration of a pulp-dentin complex without inducing inflammation, provided that 2–4 mm of apical pulp tissue remains vital in teeth diagnosed with irreversible pulpitis. Importantly, while the control group with apical pulp retention alone also showed some regenerative capacity, it was accompanied by persistent intracanal inflammation and disorganized tissue architecture, underscoring the immunomodulatory and pro-regenerative dual role of hUCMSCs (Sabeti et al., 2024).

Shifting the focus to the crucial role of angiogenesis, Angelopoulos et al. compared DPSCs and hUCMSCs in PPP scaffolds on human dentin discs implanted in mice. Both cell types enhanced hemoglobin content, promoted new blood vessel formation versus PPP-only controls, and induced new dentin deposition, confirming PPP as a feasible delivery vehicle and supporting both cell sources for regenerative endodontics (Angelopoulos et al., 2022).

Collectively, these studies demonstrate that hUCMSCs, when combined with functionally tailored scaffolds, hold versatile and promising utility for dental pulp and dentin regeneration.

##### 4.3.1.2 Optimization of cellular strategies

In addition to investigating different scaffold materials, researchers have also sought to optimize the cellular composition of tissue-engineered constructs. Beyond the use of hUCMSCs alone, co-culture strategies have shown significant promise in enhancing regenerative outcomes (Wu et al., 2025).

Based on the finding that VEGF induction triggers the expression of functional endothelial markers in hUCMSCs, Zhang et al. established a “self-sourced” co-culture system in which VEGF-induced hUCMSCs (V-hUCMSCs) were co-transplanted with untreated hUCMSCs. *In vivo* Matrigel plug assays demonstrated that co-culture grafts formed robust vessel-like structures. This pro-angiogenic effect may be attributed to hUCMSCs facilitating the upregulation of long non-coding RNA HIF1A-AS2 expression in V-hUCMSCs, thereby activating the Hypoxia-inducible factor 1 (HIF-1) signaling pathway and ultimately enhancing the angiogenic capacity of V-hUCMSCs (Zhang et al., 2020a). To achieve a more optimized angiogenic effect, Zhang et al. further investigated the co-culture of hUCMSCs with primary HUVECs, which demonstrated that a co-culture ratio of hUCMSCs to HUVECs at 1:5 yielded the most significant pro-angiogenic outcome. Both *in vitro* Matrigel assays and *in vivo* Matrigel plug assays revealed the formation of a more extensive and stable vascular network under this condition. Mechanistic studies further implicated the activation of the HIF1A-AS2/HIF-1 signaling pathway, suggesting that hUCMSCs enhance angiogenesis in different endothelial cell types through a similar molecular mechanism (Zhang et al., 2020b). Furthermore, both studies independently evaluated autologous and heterologous co-culture systems for promoting dental pulp regeneration using a consistent *in vivo* approach. In each case, co-cultured cells (hUCMSCs with V-hUCMSCs or hUCMSCs with HUVECs, respectively) were encapsulated in type I collagen scaffolds, injected into human root segments, and implanted subcutaneously in immunodeficient mice to mimic a pulp-regeneration environment. Results demonstrated that all cell-transplantation groups regenerated pulp-like tissue within root canals, with co-culture groups exhibiting enhanced extracellular matrix formation and greater collagen deposition compared to monoculture controls (Zhang et al., 2020a; 2020b).

In another study focusing on odontogenic differentiation, Huang et al. co-cultured BMP-2-pre-induced human dental pulp cells (hDPCs) with hUCMSCs at a 1:1 ratio in a RADA16-I hydrogel scaffold, which supported both cell types. This co-culture enhanced cell proliferation, alkaline phosphatase activity, and mineralized nodule formation versus monocultures, an effect attributed to paracrine signaling and gap junction-mediated cell-cell communication from hDPCs to hUCMSCs (Huang et al., 2020).

These findings underscore the promise of combining differentially primed cell populations in a three-dimensional co-culture system to amplify odontogenic outcomes for pulp-dentin regeneration.

##### 4.3.1.3 Progress of clinical trials

The promising preclinical outcomes of hUCMSC-based strategies have catalyzed their translation into early-phase clinical investigations for dentin-pulp regeneration.

Brizuela et al. reported two clinical cases of mature teeth with pulp necrosis and periapical periodontitis treated with PPP-encapsulated hUCMSCs in regenerative endodontic procedures. After 5 years, both cases showed satisfactory healing and pulp revitalization (Brizuela et al., 2024). In an earlier phase I/II randomized controlled trial, the same group implanted hUCMSCs in a PPP matrix into root canals of 18 patients. At 12 months, all achieved clinical success, demonstrating the safety and efficacy of this approach for dentin-pulp regeneration and periapical tissue preservation (Brizuela et al., 2020).

In a more complex case—a mature permanent tooth with apical periodontitis and root perforation—Cordero et al. used allogeneic hUCMSCs in a PPP matrix. Over 12 months, the patient remained asymptomatic, with CBCT showing periapical lesion regression and dentin bridge formation, demonstrating the feasibility of this protocol for complex iatrogenic scenarios (Cordero et al., 2020).

Together, these studies, progressing from initial case reports through a controlled trial to a complex case report, constitute an evolving clinical evidence chain that underscores the translational feasibility and growing therapeutic promise of hUCMSC-scaffold approaches in regenerative endodontics.

#### 4.3.2 Exosome-functionalized scaffolds for pulp-dentin regeneration

In the field of dental pulp and dentin regeneration, researchers have also focused on combining hUCMSC-Exos with scaffolds as a promising therapeutic strategy. One study demonstrated that hUCMSC-Exos exhibited a more potent effect than human dental pulp stem cell-derived exosomes (hDPSC-Exos) in suppressing inflammation and promoting the proliferation and migration of human dental pulp stem cells (hDPSCs), indicating the feasibility of hUCMSC-Exos in dental pulp treatment (Zeng J et al., 2022).

Increasing evidence suggests that the tissue-specific microenvironment can influence the function of exosomes secreted by mesenchymal stem cells (MSCs), and that lipopolysaccharide (LPS) pretreatment significantly enhances the paracrine protective, regenerative, and repair capacities of MSCs(Ti et al., 2015). Zeng et al. investigated the use of a collagen sponge/self-assembling peptide nanofiber scaffold (CS/SAPNS; note that CS here refers to collagen sponge, not chitosan) to deliver LPS-pretreated hUCMSC-derived exosomes (LPS-hUCMSC-Exos, termed LPS-hUCMSC-sEVs in the original study) for regenerating the dentin-pulp complex. Their results showed that LPS-hUCMSC-Exos delivered via the CS/SAPNS scaffold significantly enhanced the proliferation, migration, osteo/odontogenic differentiation, and secretion of angiogenic factors in hDPSCs. These effects suggest that this CS/SAPNS/Exos composite may facilitate the formation of a functional dentin-pulp complex and promote microangiogenesis, processes closely associated with Nuclear Factor Kappa B (NF-κB) pathway activation (Zeng et al., 2024). Collectively, these findings support the potential of LPS-hUCMSC-Exos as a promising therapeutic strategy for dentin-pulp regeneration.

Furthermore, some researchers have also conducted relevant early clinical explorations. In a clinical case of irreversible pulpitis, Jafari et al. applied hUCMSC-Exos mixed with chitosan into the root canal of a pulpectomized tooth. Over 24 weeks of follow-up, the patient remained stable with no signs of infection. Radiographic and CBCT imaging revealed progressive periapical healing and good integration of the exosome-chitosan mixture without adverse reactions. This study provides preliminary clinical support for hUCMSC-Exos as a promising cell-free alternative in dental pulp regeneration (Jafari et al., 2025).

## 5 Conclusion and future perspectives

In conclusion, this review has summarized the synergistic potential of hUCMSC-based therapeutic approaches and advanced biological scaffolds as a promising regenerative strategy for oral and maxillofacial tissue engineering. The unique attributes of hUCMSCs, including their multi-lineage differentiation capacity, immunomodulatory and paracrine functions, with exosomes serving as key mediators, coupled with low immunogenicity and ethical acceptability, position them as a practical alternative to traditional stem cell sources. Concurrently, advances in the design and application of scaffold materials, such as bioceramics, biologically derived scaffolds, polymers, and composites, have provided essential three-dimensional niches that enhance the delivery, retention, and efficacy of these therapeutic agents. As evidenced in bone/cartilage regeneration, periodontal repair, and pulp-dentin complex regeneration, the strategic combination of these hUCMSC-based strategies with tailored scaffolds has yielded promising preclinical and emerging clinical outcomes, moving towards functional restoration, although true physiological reconstruction remains a long-term goal.

To advance these promising strategies from preclinical validation toward clinical reality, several directions can be considered for future research. First, regarding the optimization of scaffold materials, future designs can evolve from structurally supportive matrices to dynamic, bio-instructive systems that more effectively mimic the anisotropic and viscoelastic properties of native oral tissues (e.g., periodontal ligament, TMJ disc) to achieve biomechanical compatibility (Fidalgo et al., 2025; Kosewski et al., 2025). For targeted applications, scaffolds can be engineered with specific functionalities, such as intrinsic antibacterial properties for infectious defects or immunomodulatory capabilities for hyperglycemic microenvironments (Su et al., 2025; Wang B. et al., 2025; Zare et al., 2025). Beyond material composition and surface functionalization, scaffold microarchitecture itself represents a further design variable whose systematic investigation remains limited in craniomaxillofacial regeneration (Baggio et al., 2025). Parameters such as pore size, porosity, and interconnectivity may differ across material classes and tissue models. Other scaffold properties, including mechanical strength, degradation behavior, and vascularization capacity, are also reported inconsistently across the studies reviewed here. Future studies could therefore adopt gradient-based designs to examine how individual microarchitectural parameters influence regeneration in specific craniomaxillofacial tissues. Second, in the optimization of cellular strategies, a deeper exploration of the specific molecular mechanisms governing hUCMSCs differentiation would be valuable (Huang et al., 2025). Future efforts could employ techniques such as genetic editing to modify cellular properties or fine-tune their differentiation programs, thereby enhancing their therapeutic utility in complex tissue regeneration (Chen R. et al., 2025). For instance, precise targeting could direct hUCMSC fate toward osteogenic, chondrogenic, or odontogenic lineages. Additionally, standardizing induction protocols and optimizing co-culture systems, such as defining optimal cell ratios, timing, and spatial organization within scaffolds, may be key to maximizing synergistic outcomes (Deszcz and Bar, 2025). Third, the advancement of exosome-scaffold cell-free therapeutics would benefit from the development of scaffold materials that not only preserve exosome bioactivity but also enable their spatiotemporal controlled release (Moradi et al., 2023). Extracellular vesicle-biomaterial coupling can be broadly categorized into physical entrapment, non-covalent tethering, and covalent immobilization (Stiliyanov-Atanasov et al., 2025). In the studies reviewed here, hUCMSC-Exos were loaded predominantly through passive adsorption or physical entrapment, whereas covalent immobilization was not employed. Covalent immobilization has been shown to trigger more robust host cell infiltration and angiogenic responses than non-immobilized exosomes at substantially lower doses (Stiliyanov-Atanasov et al., 2025). Expanding the loading strategies for hUCMSC-Exos toward covalent immobilization may therefore represent a promising direction for enhancing their therapeutic durability. In parallel, engineering exosomes through specific preconditioning or cargo loading to enhance their therapeutic potency represents a promising direction for next-generation biomaterial design (Chen M et al., 2025; Shang et al., 2026).

To further advance clinical translation, several challenges may warrant further exploration. The current lack of standardized protocols for hUCMSCs expansion and characterization remains a barrier to clinical translation (Mebarki et al., 2021). Similar challenges apply to scaffold manufacturing, as illustrated by the reproducibility issues of natural polymer-based scaffolds (Duceac and Coseri, 2022) and the manufacturing reproducibility and standardization hurdles reported for 3D-printed bone scaffolds (Tang et al., 2025). Furthermore, a deeper understanding of the long-term biosafety, fate, and mechanistic interactions between hUCMSCs and scaffolds within the complex oral microenvironment warrants further investigation (Shaikh et al., 2022). Finally, cost-effectiveness analyses may help ensure that these advanced therapies are economically viable and accessible for broad clinical adoption.

By addressing these interconnected challenges, the combination of hUCMSCs and advanced scaffolds may help translate preclinical evidence into clinical therapies for complex oral and maxillofacial reconstruction.
